# Uprooted Phylogenetic Networks

**DOI:** 10.1007/s11538-017-0318-x

**Published:** 2017-07-31

**Authors:** P. Gambette, K. T. Huber, G. E. Scholz

**Affiliations:** 1 LIGM (UMR 8049), UPEM, CNRS, ESIEE, ENPC, Université Paris-Est, 77454 Marne-la-Vallée, France; 2 School of Computing Sciences, University of East Anglia, Norwich, UK

**Keywords:** Phylogenetic network, Buneman graph, Circular split system, Closure, Median network, PC-trees, 92D15, 92B10

## Abstract

The need for structures capable of accommodating complex evolutionary signals such as those found in, for example, wheat has fueled research into phylogenetic networks. Such structures generalize the standard model of a phylogenetic tree by also allowing for cycles and have been introduced in rooted and unrooted form. In contrast to phylogenetic trees or their unrooted versions, rooted phylogenetic networks are notoriously difficult to understand. To help alleviate this, recent work on them has also centered on their “uprooted” versions. By focusing on such graphs and the combinatorial concept of a split system which underpins an unrooted phylogenetic network, we show that not only can a so-called (uprooted) 1-nested network *N* be obtained from the Buneman graph (sometimes also called a median network) associated with the split system $\Sigma (N)$ induced on the set of leaves of *N* but also that that graph is, in a well-defined sense, optimal. Along the way, we establish the 1-nested analogue of the fundamental “splits equivalence theorem” for phylogenetic trees and characterize maximal circular split systems.

## Introduction

**Fig. 1:**
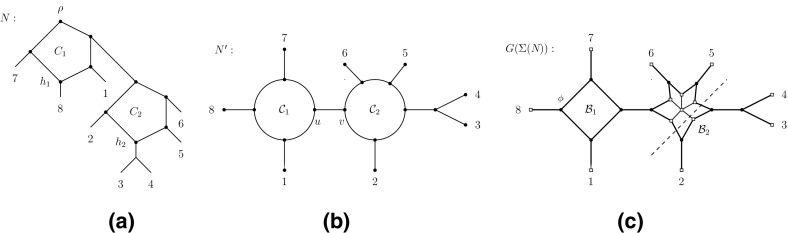
**a** A phylogenetic network *N* on $X=\{1,\ldots ,8\}$. The root is labeled by $\rho$, all edges are directed downwards, and vertices $h_1$ and $h_2$ represent hypothesized reticulate evolutionary events. **b** The uprooted version $N':=U(N)$ of *N*. **c** An unrooted phylogenetic network on *X* in the form of the Buneman graph $G(\Sigma (N))$ on the split system $\Sigma (N)$ induced by $N'$. The *dashed line* indicates the split 234|15678—see Sect. 2 for details

A widely accepted evolutionary scenario for some economically important crop plants such as wheat is that their evolution has been shaped by complex reticulate processes (Marcussen 2014). The need for structures capable of representing the telltale signs left behind by such processes has fueled research into phylogenetic networks. In the rooted case, these are generally based on the concept of a rooted directed acyclic graph whose leaf set is a pre-given set *X* of taxa (e. g.  species). Such structures have, however, turned out to be notoriously difficult to come to grips with from a combinatorial point of view (see, e.g., the graduate text books Gusfield 2014; Huson et al. 2010). Reflecting this, research into rooted phylogenetic networks has recently also centered on their “uprooted” versions (see ,e.g., Gambette and Huber 2012; Huber et al 2015; van Iersel et al. 2016; Francis et al 2017 and Fig. 1 for an example). These graphs have turned out to be more amenable to a combinatorial analysis and, at the same time, are still of interest to evolutionary biologists since they provide insights into the number of non-treelike evolutionary events undergone by a taxa set.

From a formal point of view, the uprooted version *U*(*N*) of a phylogenetic network *N* is a connected (undirected) graph with leaf set *X* such that no vertex has degree two and every cycle has length at least four. We therefore define an *uprooted (phylogenetic) network* to be a graph with these properties. In this context, it should be noted that—although related to the notion of an unrooted phylogenetic network—uprooted networks are, in general, not unrooted phylogenetic networks as they might contain cycles of odd length whereas this is not possible for the latter (see, e.g., Fig. 1b, c for an illustration of this difference).

Despite their attractiveness to evolutionary biology, it is, however, unclear how to directly construct an uprooted phylogenetic network from real data, i.e., without first constructing a (rooted) phylogenetic network and then ignoring the direction of its edges and suppressing its root. To address this, we focus on the special case that the uprooted phylogenetic network *N* is *1-nested*, that is, no two cycles in *N* share an edge (see, e.g., Rossello and Valiente 2009; Huber and Moulton 2013 for rooted versions of such networks). These types of networks allow for limited interaction between reticulate evolutionary events and, as we shall see, have attractive combinatorial properties. Calling a bipartition of *X* a *split* of *X* and a collection of splits of *X* a *split system (on X)*, it is straightforward to see that any 1-nested network *N* induces a split system $\Sigma (N)$ on its leaf set *X* by taking minimal cuts. Starting from $\Sigma (N)$, we show in Theorems 3 and 5 that the Buneman graph $G(\Sigma (N))$ associated with $\Sigma (N)$ can be used to uniquely recover *N* (up to isomorphism and a mild condition) in polynomial time and that it is optimal. These graphs are certain types of unrooted phylogenetic networks and are defined for a *split system* $\Sigma$ on *X* as follows (see, e.g., Sect. 2 for some basic properties).

### Definition 1

The *Buneman graph* $G(\Sigma )$ associated with $\Sigma$ is the graph whose vertex set $V(\Sigma )$ is the set of all maps $\phi :\Sigma \rightarrow {\mathcal {P}}(X)$ from $\Sigma$ into the powerset ${\mathcal {P}}(X)$ of *X* such that $\phi (S)\in S$ holds for all $S\in \Sigma$ and $\phi (S)\cap \phi (S')\not =\emptyset$, for all $S,S'\in \Sigma$ and whose edge set $E(\Sigma )$ is the set of all $\{\phi ,\phi '\}\in {V(\Sigma )\atopwithdelims ()2}$ for which the size of the symmetric difference $\Delta (\phi ,\phi ')$ of $Im(\phi )$ and $Im(\phi ')$ has size one.

For example for the network $N'$ pictured in Fig. 1b, the vertex of $G(\Sigma (N'))$ marked $\phi$ in Fig. 1c, and $S\in \Sigma (N')$, we have $\phi (S)=X-\{8\}$ if $S=\{\{8\},X-\{8\}\}$ and $\phi (S)=A$ otherwise where $8\in A\in S$.

As we shall see in Sect. 4, the split system induced by a 1-nested network is always circular (see Sect. 2 for a definition and Gambette et al 2012 for the treatment of a special case). This property is particularly attractive in a phylogenetics context as it guarantees that any such split system is representable in the plane in terms of an unrooted phylogenetic networks without crossing edges. Inspired by this, we characterize maximal circular split systems in Theorem 2. As a consequence, we obtain in Corollary 2 the 1-nested analogue of the fundamental “splits equivalence theorem” for phylogenetic trees (Semple and Steel 2003, Theorem 3.1.4). That theorem characterizes split systems $\Sigma$ on *X* for which there exists a unique (up to isomorphism) unrooted phylogenetic tree *T* on *X* such that $\Sigma =\Sigma (T)$, thereby providing a combinatorial description for such trees.

The outline of the paper is as follows. In the next section, we introduce relevant basic terminology and present some first insights. In Sect. 3, we state a closure rule for split systems which underpins our key tool: the $\mathcal I$-intersection closure of a split system. Intriguingly, our rule also appeared in the guise of Property (C1) in Dinitz and Nutov (1997) where it was used to characterize cactus models in terms of certain bisection families (i.e., certain split systems). Cactus models are closely related to rooted versions of *level-1* phylogenetic networks (i.e., the requirement that no two cycles share an edge is strengthened to the requirement that no two cycles share a vertex). However, cactus models are not rooted phylogenetic networks in the usual sense. We clarify the relationship between them first and then present a characterization of split systems that can be displayed by 1-nested networks. In Sect. 4, we establish Theorems 2, 3 and Corollary 2. Using insights into the structure of the Buneman graph presented in Dress et al. (2011), we establish Theorem 5 in Sect. 5. We conclude with some open problems in Sect. 6.

## Preliminaries

In this section, we present relevant basic definitions concerning split systems and phylogenetic networks. Also, we clarify how rooted and unrooted phylogenetic networks represent a split. Throughout the paper, we assume that *X* is a finite set with $n\ge 3$ elements. Also, unless stated otherwise, split systems are assumed to be non-empty.

### Uprooted Phylogenetic Networks

Suppose *G* is a simple connected graph. A *cut vertex* of *G* is a vertex whose deletion along with its incident edges disconnects *G*. A *cut edge* of *G* is an edge whose removal disconnects *G*. We call a cut edge *trivial* if it is incident to a *leaf* *v* of *G*, that is, the degree of *v* is one. Similarly, for rooted phylogenetic networks, we call an uprooted phylogenetic network *N* *simple* if all cut edges of *N* are trivial and we say that two uprooted phylogenetic networks *N* and $N'$ on *X* are *isomorphic* if there exists a graph isomorphism between *N* and $N'$ that is the identity on *X*. To keep terminology at bay, we shall from now refer to an uprooted phylogenetic network as just a phylogenetic network, or a network, for short.

### Splits and Split Systems

For all subsets $A\subseteq X$, we put $\bar{A}=X-A$. For $A\subseteq X$ a proper subset of *X*, we call the pair $\{A, \bar{A}\}$ a *split* of *X*. For convenience, we denote a split $S=\{A,B\}$ of *X* also by *A*|*B* or, equivalently, by *B*|*A*. If $A=\{x_1,\ldots ,x_k\}$ and $B=\{x_{k+1},\ldots , x_n\}$ for some $1\le k\le n-1$ we write $x_1\ldots x_k|x_{k+1}\ldots x_n$ rather than $\{x_1,\ldots ,x_k\}|\{x_{k+1},\ldots , x_n\}$. Furthermore, for all elements $x\in X$ and all splits *S* of *X*, we denote by *S*(*x*) the element of *S* that contains *x*. The *size* of a split *A*|*B* is defined as $\min \{|A|,|B|\}$. We call a split *S* *trivial* if its size is one.

Two distinct splits $S_1$ and $S_2$ of *X* are called *compatible* if there exists some $A_1\in S_1$ and some $A_2\in S_2$ such that $A_2\subsetneq A_1$ and *incompatible* otherwise. More generally, a split system $\Sigma$ on *X* is called *compatible* if any two distinct splits in $\Sigma$ are compatible and *incompatible* otherwise.

Suppose $x_1,x_2,\ldots ,x_n,x_{n+1}:=x_1$ is a circular ordering of the elements of *X*. For all $i,j\in \{1,\ldots ,n\}$ with $i\le j$ (where we take indices modulo *n*), we call the subsequence $x_i,x_{i+1},\ldots , x_j$ the *interval* from $x_i$ to $x_j$ and denote it by $[x_i, x_j]$. We say that a split system $\Sigma$ on *X* is *circular* if there exists a circular ordering $x_1, x_2,\ldots , x_n, x_{n+1}:=x_1$ of the elements of *X* such that for every split $S=A|B\in \Sigma$ there exists an $i,j\in \{1,\ldots ,n\}$ such that $A=[x_i,x_j]$ and $B=[x_{j+1}, x_{i-1}]$. Note that there are $(n-1)!$ circular orderings for *X* and that a circular split system on *X* has size at most $n(n-1)/2$.

### Displaying Splits

As it turns out, both uprooted networks and Buneman graphs display a split system but the way they do this is fundamentally different. In this section, we first present formal definitions for each case and then an example illustrating this difference.

#### Uprooted Networks

Suppose *G* is a graph. Then we call a set *E* of edges of *G* a *cut* of *G* if the deletion of all edges in *E* disconnects *G*. For *N* a phylogenetic network on *X* and $S=A|B$ a split of *X*, we say that *S* is *displayed* by *N* if there exists a (set inclusion) minimal cut $E_S$ of *N* resulting in two connected components one of whose set of leaves is *A* and the other is *B*. The *multiplicity* of a split $S\in \Sigma (N)$ is the number of distinct minimal cuts of *N* that induce *S*. More generally, we say that a split system $\Sigma$ is *displayed* by *N* if every split of $\Sigma$ is displayed by *N*, that is, $\Sigma \subseteq \Sigma (N)$. Also, we say that a split $S\in \Sigma (N)$ is *displayed by a cycle C* of *N* if $E_S$ is contained in the edge set of *C*.

Note that in case *N* is a 1-nested network and $S\in \Sigma (N)$ we have $|E_S|\in \{1,2\}$. Also, if $e=\{u,v\}$ is the unique element in $E_S$ and neither *u* nor *v* is contained in a cycle of *N*, then *e* must be a cut edge of *N* and the multiplicity of the split $S_e$ induced by deleting *e* is one. Moreover, if $e=\{u,v\}$ is a cut edge of *N* where *u* or *v* is contained in a cycle *C* of *N*, say *u*, then $S_e$ is also induced by deleting the edges of *C* incident with *u*. Thus, the number of distinct minimal cuts in a 1-nested network inducing a given split in $\Sigma (N)$ can either be one, two, or three. Furthermore, the split system $\Sigma (N)$ induced by a 1-nested network *N* on *X* is the same as the one induced by the resolution of *N* to a level-1 network by repeatedly applying the following two replacement operations (and their converses which we denote by (R1$'$) and (R2$'$), respectively):

1. a vertex *v* of a cycle *C* of *N* incident with $l\ge 2$ edges $e_1,\ldots ,e_l$ not contained in *C* is replaced by an edge one of whose vertices is *v* and the other is incident with $e_1,\ldots ,e_l$, and
2. a cut vertex *v* shared by two cycles $C_1$ and $C_2$ is replaced by a cut edge one of whose vertices is contained in $C_1$ and the other in $C_2$.

However, the multi-sets of splits induced by both networks are clearly different. We call the vertex *v* in (R1) or (R2) *partially resolved*. More generally, we call a 1-nested network $N'$ a *partial resolution* of a 1-nested network *N* if $N'$ can be obtained from *N* by partially resolving vertices of *N*. Moreover, we call a partial resolution $N'$ of *N* a *maximal partial resolution* of *N* if the only way to obtain a partial resolution of $N'$ is to apply (R1$'$) or (R2$'$). In this case, we also call $N'$ *maximal partially resolved*. Finally, we call a split *S* of multiplicity two or more in a maximal partial resolution of *N* an *m-split* of *N* (or more precisely of *C* if *C* is the cycle of *N* that displays *S*).

**Fig. 2:**
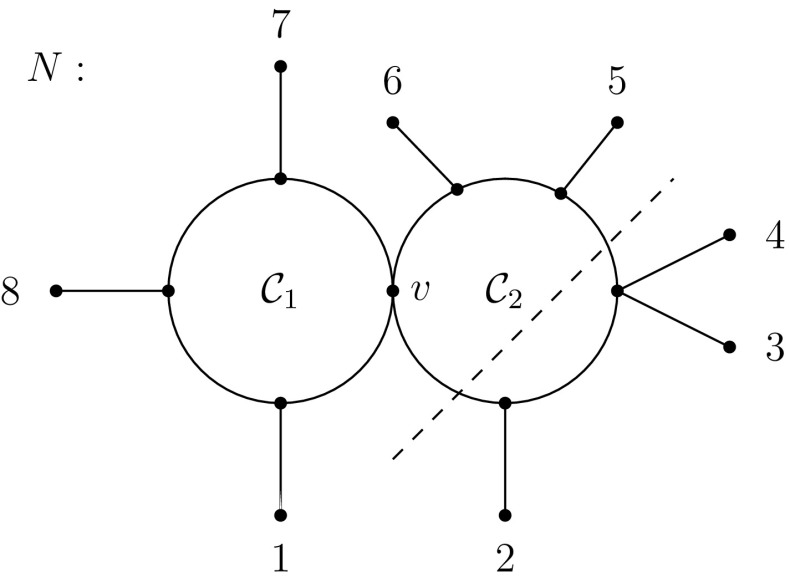
A 1-nested network for which the induced split system is $\Sigma (N')$ where $N'$ is the uprooted network in Fig. 1b. As in Fig. 1c, the dashed line indicates the split 234|15678

To illustrate some of these definitions, consider the 1-nested network *N* on $X=\{1,\ldots , 8\}$ depicted in Fig. 2. Then the split 234|15678 is displayed by *N* as it is induced by deleting the pair of edges crossed by the dashed line. The splits $7|X-\{7\}$, $8|X-\{8\}$, $1|X-\{1\}$ and 781|23456 are m-splits for the cycle $C_1$ of *N*. Furthermore, $N'$ is a partial resolution of *N* and the multi-set of splits induced by *N* only contains splits of multiplicity one or two.

#### Buneman Graph

Buneman graphs are sometimes also called median networks and have been shown to be isometric subgraphs of hypercubes (see, e.g., Dress et al. 2012). They have appeared in the literature under various guises such as co-pair hypergraphs (Barthelemy 1989, 1991) and have been studied in terms of median algebras (Bandelt and Hedliková 1983), 1-skeletons of CAT(0) cubical complexes (Bandelt and Chepoi 1996), retracts of hypercubes (Bandelt 1984), tight spans of metric spaces [see, e.g., Dress et al. 2002 and also the more recent text book (Dress et al. 2012) and the references therein], and S2 binary convexities (van de Vel 2016) (see, e.g., Klavzar and Mulder 1999 for a review of median graphs). Split systems induced by 1-nested network on some set *X* clearly contain all trivial splits on *X*. As it turns out for any split system $\Sigma$ on *X* that contains all trivial splits on *X* the set of degree one vertices in $G(\Sigma )$ is precisely the set of Kuratowski maps associated with *X*, where for some $x\in X$, we refer to the unique map $\phi _x:\Sigma \rightarrow \mathcal P(X)$ defined by putting $\phi _x(S)= S(x)$ as the *Kuratowski map* associated with *x*. Following standard practice, we identify the set of degree 1 vertices of $G(\Sigma )$ with *X*.

Suppose for the following that $\Sigma$ is a split system on *X*. By abuse of terminology, we denote for all edges $e=\{\phi ,\phi '\}\in E(\Sigma )$, the unique split in $\Delta (\phi ,\phi ')$ by $S_e$. We say that a split $S=A|B$ of *X* is *Bu-displayed* by $G(\Sigma )$ if there exists a “ladder” $E'$ of parallel edges in $G(\Sigma )$ whose deletion disconnects $G(\Sigma )$ into two connected components one of whose vertex sets contains *A* and the other *B* (see, e.g., Dress et al. 2012, Lemma 4.5) for details where Bu-displayed is called displayed. Note that every split that is Bu-displayed by $G(\Sigma )$ is also a minimal cut of $G(\Sigma )$ and thus displayed by $G(\Sigma )$. However, the converse need not hold.

To illustrate these definitions, let $X=\{1,\ldots ,8\}$ and consider again the split system $\Sigma$ displayed by the 1-nested network depicted in Fig. 2. Then the Buneman graph $G(\Sigma )$ associated with $\Sigma$ is depicted in Fig. 1c, and the deletion of the “ladder” of edges crossed by the dashed line generates the split 234|15678. Thus, that split is Bu-displayed by $G(\Sigma )$.

## Characterizing of 1-Nested Networks in Terms of ${\mathcal {I}}$-Intersections

In this section, we introduce and study the ${\mathcal {I}}$-intersection closure of a split system. Intriguingly, the rule underpinning it appeared in the guise of Property (C1) in Dinitz and Nutov (1997) where it was used to characterize certain graph models called cactus models (Dinitz and Nutov 1997, Theorem 3.1) in terms of bisection families of a set. For *F* a bisection family (i.e., a split system) defined on *X*, a *graph model* is an ordered 3-tuple $\Omega =(G,\phi , {\mathcal {F}})$ where *G* is a graph, ${\mathcal {F}}$ is a family of bisections of the vertex set *V*(*G*) of *G* and $\phi$ is a map from *X* to *V*(*G*) such that $\phi ^{-1}({\mathcal {F}})=F$. In case *G* is a cactus, $\Omega$ is called a *cactus model*. To define a cactus, suppose *H* is a graph. Then *H* is called *2-connected* if, after deletion of any of its vertices, it remains connected or is an isolated vertex (Dress et al. 2011). Furthermore, a subgraph of *H* is called a *block* of *H* if it is a maximal 2-connected component of *H*. Then a cactus is a connected graph such that each of its blocks is either an edge or a cycle.

Within the context of this paper, it is important to note that the image of $\phi$ is contained in *V*(*G*) (rather than equal to the leaf set of *G*). Thus, *G* may contain interior vertices that are mapped by $\phi$ to one (or more!) elements in *X* and also interior vertices that have degree two—see, e.g., Brandes and Cornelsen (2010), Fig. 5. Both situations are not allowed for rooted phylogenetic networks (see, e.g., Huson et al. 2010, p. 138) and, thus, also uprooted ones. This suggests that cactus models are more like direct generalizations of *X*-trees (as defined in Semple and Steel 2003) than of phylogenetic trees. Thus, rather than deriving our characterization of 1-nested networks *N* in terms of split systems as a corollary of Dinitz and Nutov (1997), Theorem 3.1, we present a more direct, alternative proof (Theorem 1). As we shall see, our proof heavily relies on the fact that $\phi (X)$ is precisely the leaf set of *N*. In conjunction with Sect. 5, this implies that our proof is constructive in nature.

We start with introducing the concept of an intersection between splits. Suppose $S_1$ and $S_2$ are two distinct splits of *X* and $A_i \in S_i$, $i=1,2$, such that $A_1 \cap A_2 \ne \emptyset$. Then we call the split $A_1 \cap A_2 | \bar{A}_1 \cup \bar{A}_2$ of *X* associated with $\{S_1,S_2\}$ an *intersection of* $S_1$ *and* $S_2$ (*with respect to* $A_1$ *and* $A_2$). We denote the set of all splits obtained by taking intersections of $S_1$ and $S_2$ by $int(S_1,S_2)$ and write $int(S_1,S_2)$ rather than $int(\{S_1,S_2\})$. Furthermore, if $S_1$ and $S_2$ are incompatible, then we refer to the intersection of $S_1$ and $S_2$ as *incompatible intersection*, or ${{\mathcal {I}}-intersection}$ for short, and denote it by $\iota (S_1,S_2)$ rather than $int(S_1,S_2)$.

**Fig. 3:**
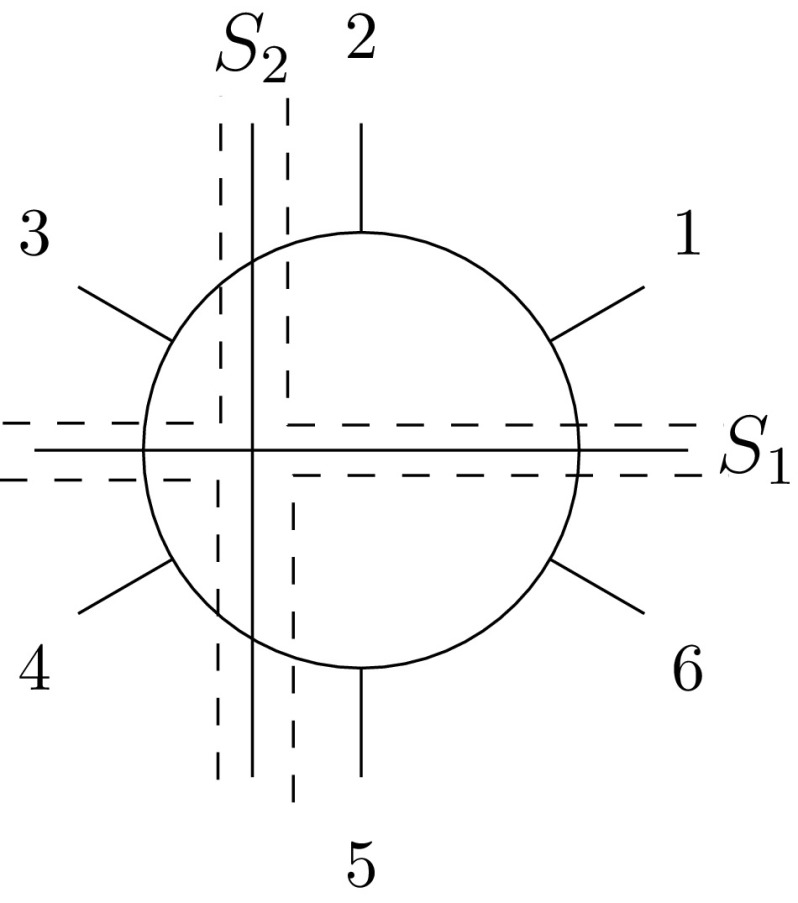
For a simple level-1 network on $\{1,\ldots , 6\}$, we depict the splits $S_1$ and $S_2$ in terms of *two straight bold lines* and the *four splits* that make up $\iota (S_1,S_2)$ in terms of *four dashed lines*

Clearly, if $S_1$ and $S_2$ are compatible, then $|int(S_1,S_2)|=3$ and $S_1,S_2\in int(S_1,S_2)$. However, if $S_1$ and $S_2$ are incompatible, then $\iota (S_1,S_2)$ is compatible and of size four, $S_1,S_2\not \in \iota (S_1,S_2)$, and every split in $\iota (S_1,S_2)$ is compatible with $S_1$ and $S_2$. See Fig. 3 for an illustration.

Figure 3 shows that every split in $\iota (S_1,S_2)$ is displayed by the same cycle that displays $S_1$ and $S_2$. Establishing that this is indeed the case is the purpose of Proposition 1. To state it in its full generality, we next associate with a split system $\Sigma$ of *X* the *intersection closure* $Int(\Sigma )$ of $\Sigma$, that is, $Int(\Sigma )$ is a (set inclusion) minimal split system that contains $\Sigma$ and is closed by intersection. For example, for $\Sigma =\{12|345, 23|451\}$ we have $Int(\Sigma )=\Sigma \cup \{1|2345, 2|3451, 3|4512, 13|452, 123|45\}$.

We start our analysis of $Int(\Sigma )$ with remarking that $Int(\Sigma )$ is indeed a closure, that is, $Int(\Sigma )$ trivially satisfies the following three properties

1. $$\Sigma \subseteq Int(\Sigma )$$.
2. $$Int(Int(\Sigma ))= Int(\Sigma )$$.
3. If $\Sigma '$ is a split system on *X* for which $\Sigma \subseteq \Sigma '$ holds then $Int(\Sigma )\subseteq Int(\Sigma ')$.

The next lemma implies that the intersection closure of a split system is well defined.

### Lemma 1

Suppose $\Sigma$ is a split system on *X* and $\Sigma '$ is a further (set inclusion) minimal superset of $\Sigma$ that is closed by intersection. Then $\Sigma ' =Int(\Sigma )$ must hold.

### Proof

Since $\Sigma '$ contains $\Sigma$ and is intersection closed, we can obtain $\Sigma '$ via a (finite) sequence $\Sigma =\Sigma _0\subsetneq \Sigma _1\subsetneq \Sigma _2\subsetneq \cdots \subsetneq \Sigma _k=\Sigma '$, $k\ge 1$, of split systems $\Sigma _i$ such that, for all $1\le i\le k$, $\Sigma _{i}:=\Sigma _{i-1}\cup \iota (P_{i})$ where $P_{i}$ is a 2-set contained in $\Sigma _{i-1}$ and $\iota (P_{i})$ is not contained in $\Sigma _{i-1}$. We show by induction on *i* that $\Sigma _{i}\subseteq Int(\Sigma )$ holds.

Clearly, if $i=0$ then $\Sigma _0=\Sigma$ is contained in $Int(\Sigma )$. So assume that $\Sigma _{i}\subseteq Int(\Sigma )$ holds for all $1\le i\le r$, for some $1\le r\le k$, and that $\Sigma _r$ is obtained from $\Sigma _{r-1}$ by intersection of two splits $S_1, S_2\in \Sigma _{r-1}$. Since, by induction hypothesis, $\Sigma _{r-1}\subseteq Int(\Sigma )$, it follows that $S_1$ and $S_2$ are contained in $Int(\Sigma )$. Since $Int(\Sigma )$ is intersection closed, $\iota (S_1,S_2)\subseteq Int(\Sigma )$ follows. Hence, $\Sigma _r=\Sigma _{r-1}\cup \iota (S_1,S_2)\subseteq Int(\Sigma )$, as required. By induction, it now follows that $\Sigma '\subseteq Int(\Sigma )$. Reversing the roles of $\Sigma '$ and $Int(\Sigma )$ in the previous argument implies that $Int(\Sigma )\subseteq \Sigma '$ holds too which implies $\Sigma '= Int(\Sigma )$. $\square$

We remark in passing that similar arguments as the ones used in the proof of Lemma 1 also imply that the ${\mathcal {I}}$-intersection closed (set inclusion) minimal superset ${\mathcal {I}}(\Sigma )$ of a split system $\Sigma$ is also well defined [and obviously satisfies Properties (P1)—(P3)]. We will refer to ${\mathcal {I}}(\Sigma )$ as ${\mathcal {I}}$-*intersection closure of* $\Sigma$.

We next turn our attention to the ${\mathcal {I}}$-intersection closure of a split systems induced by a 1-nested network.

### Proposition 1

Suppose *N* is a 1-nested network on *X* and $S_1$ and $S_2$ are two incompatible splits contained in $\Sigma (N)$. Then $\iota (S_1,S_2)\subseteq \Sigma (N)$.

### Proof

Note first that two splits *S* and $S'$ induced by a 1-nested network are incompatible if and only if they are displayed by pairs of edges in the same cycle *C* of *N*. For $i=1,2$, let $\{e_i,e'_i\}$ denote the edge set whose deletion induces the split $S_i$. Then since $S_1$ and $S_2$ are incompatible, we have $\{e_1,e'_1\}\cap \{e_2,e'_2\}=\emptyset$ and none of the connected components of *N* obtained by deleting $e_i$ and $e'_i$ contains both $e_j$ and $e'_j$, for all $i,j\in \{1,2\}$ distinct. Without loss of generality, we may assume that when starting at edge $e_1$ and moving clockwise through *C* we first encounter $e_2$, then $e_1'$ and, finally $e_2'$ before returning to $e_1$. Then it is straightforward to see that a split in $\iota (S_1,S_2)$ is displayed by one of the edge sets $\{e_1,e_2\}$, $\{e_2,e_1'\}$, $\{e_1',e_2'\}$, and $\{e_2',e_1\}$. Thus, $\iota (S_1,S_2)\subseteq \Sigma (N)$. $\square$

Combined with the definition of the ${\mathcal {I}}$-intersection closure, we obtain the following result [see also (Kleinman et al 2013, Lemma 4.3)] for the case of statement (ii).

### Corollary 1

The following statements hold:

(i) If $\Sigma$ is a circular split system for some circular ordering of *X*, then $\mathcal {I}(\Sigma )$ is also circular for that ordering.
(ii) If *N* is a 1-nested network on *X*, then $\Sigma (N)$ is $\mathcal {I}$-intersection closed. Furthermore, *N* displays a split system $\Sigma$ on *X* if and only if *N* displays $\mathcal {I}(\Sigma )$.

The next observation is almost trivial and is used in the proof of Theorem 1.

### Lemma 2

Suppose $x\in X$ and $S_1$, $S_2$, and $S_3$ are three distinct splits of *X* such that $S_3(x)\subseteq S_1(x)$, $S_3$ and $S_2$ are compatible and $S_1$ and $S_2$ are incompatible. Then $S_3(x)\subseteq S_2(x)$.

### Proof

Since $S_2$ and $S_3$ are compatible either $S_2(x)\subseteq S_3(x)$ or $S_3(x)\subseteq S_2(x)$ or $\overline{S_3(x)}\subseteq S_2(x)$ must hold. If $S_2(x)\subseteq S_3(x)$, then $S_2(x)\subseteq S_1(x)$ which is impossible since $S_1$ and $S_2$ are incompatible. If $\overline{S_3(x)}\subseteq S_2(x)$ held, then $\emptyset \not =\overline{S_1(x)}\cap \overline{S_2(x)} \subseteq \overline{S_3(x)}\cap \overline{S_2(x)}=S_2(x)\cap \overline{S_2(x)} =\emptyset$ follows which is impossible. $\square$

For clarity of presentation, we remark that for the proof of Theorem 1, we will assume that if a given split *S* of a 1-nested network *N* has multiplicity at least two in the multi-set of splits induced by *N* then *S* is displayed by a cycle *C* of *N* (rather than by a cut edge of *N*). Furthermore, we denote the split system of *X* induced by a cycle *C* of a 1-nested network *N* on *X* by $\Sigma (C)$. Clearly, $\Sigma (C)\subseteq \Sigma (N)$ holds.

### Theorem 1

Suppose $\Sigma$ is a split system on *X* that contains all trivial splits of *X*. Then the following hold:

(i) There exists a 1-nested network *N* on *X* such that $\Sigma =\Sigma (N)$ if and only if $\Sigma$ is circular and $\mathcal {I}$-intersection closed.
(ii) A maximal partially resolved 1-nested network *N* is a level-1 network if and only if there exists no split of *X* not contained in $\Sigma (N)$ that is compatible with every split in $\Sigma (N)$.

### Proof

(i): Assume first that there exists a 1-nested network *N* on *X* such that $\Sigma =\Sigma (N)$. Then arguments similar to the ones used in Gambette et al (2012), Theorem 2 to establish that the split system induced by a level-1 network is circular imply that $\Sigma (N)$ is circular. Hence, $\Sigma$ must be circular. That $\Sigma$ is ${\mathcal {I}}$-intersection closed follows by Corollary 1(ii).

Conversely, assume that $\Sigma$ is circular and ${\mathcal {I}}$-intersection closed. Then there clearly exists a 1-nested network *N* such that $\Sigma \subseteq \Sigma (N)$. Let *N* be such that $|\Sigma (N)|$ is minimal among all 1-nested networks on *X* satisfying that set inclusion.^1^ Without loss of generality, we may assume that *N* is maximal partially resolved. We show that, in fact, $\Sigma = \Sigma (N)$ holds. Assume for contradiction that there exists a split $S_0 \in \Sigma (N) - \Sigma$. Since $\Sigma (N)$ must contain all trivial splits of *X*, it follows that $S_0$ cannot be a trivial split of *X*. In view of the remark preceding the theorem, $S_0$ is induced by either (a) deleting a cut edge $e=\{u,v\}$ of *N* and neither *u* nor *v* are contained in a cycle of *N* or (b) deleting two distinct edges of the same cycle of *N*.

**Fig. 4:**
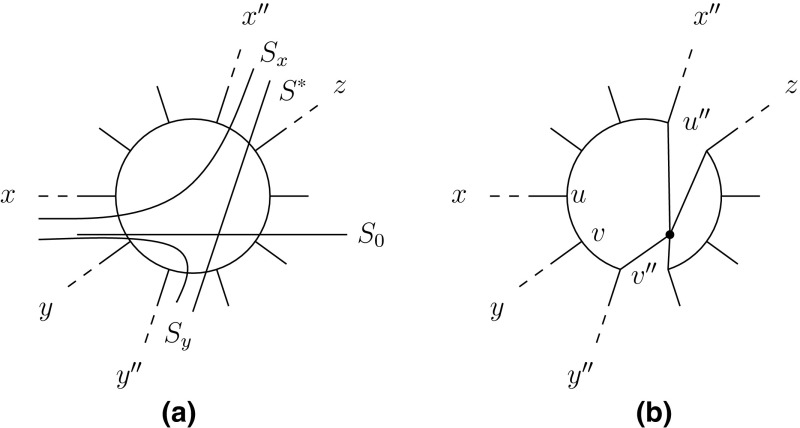
**a** An illustration of the reduction process considered in the proof of Case (a) of Theorem 1. **b** Again for that theorem, the graph $G'$ obtained from *N* by adding subdivision vertices *r* and $r'$

Assume first that Case (a) holds. Then collapsing *e* results in a 1-nested network $N'$ on *X* for which $\Sigma \subseteq \Sigma (N')$ holds. But then $|\Sigma (N')| < |\Sigma (N)|$ which is impossible in view of the choice of *N*. Thus, Case (b) must hold, that is, $S_0$ is induced by deleting two distinct edges $e=\{u,v\}$ and $e'=\{u',v'\}$ of the same cycle *C* of *N*. Let *x* and *y* be two elements of *X* for which there exists a path from *u* and *v*, respectively, which does not cross an edge of *C*. Consider the sets $\Sigma _x:=\{S \in \Sigma \cap \Sigma (C): S(x) \subseteq S_0(x) \}$, and $\Sigma _y:=\{S \in \Sigma \cap \Sigma (C): S(y) \subseteq S_0(y)\}$. If $\Sigma _x$ is non-empty then choose some $S_x\in \Sigma _x$ such that $|S_x(x)|$ is maximal among the splits contained in $\Sigma _x$. Similarly, define the split $S_y$ for $\Sigma _y$ if $\Sigma _y$ is non-empty. Otherwise, let $S_x$ be the m-split of *C* such that $S_x(x)\subseteq S_0(x)$. Similarly, let $S_y$ be the m-split of *C* such that $S_y(y)\subseteq S_0(y)$ in case $\Sigma _y$ is empty. Then, Corollary 1(ii) implies that the split

$$\begin{aligned} S^*=S_x(x) \cup S_y(y) |\overline{ S_x(x)} \cap \overline{S_y(y)} \end{aligned}$$

is contained in $\Sigma (N)$ (see Fig. 4a for an illustration).

We next show that $$S^*$$ is compatible with every split in $$\Sigma $$. To this end, we first claim that every split $$S'\in \Sigma $$ that is incompatible with $$S^*$$ must be compatible with at least one of $$S_x$$ and $$S_y$$. To see this, let $$S'\in \Sigma $$ such that $$S'$$ and $$S^*$$ are incompatible. Then $$S'$$ must be displayed by *C*. For contradiction, assume that $$S'$$ is incompatible with both of $$S_x$$ and $$S_y$$. Let $$z\in X$$ such that $$S^*(x)\not =S^*(z)$$ and let $$u''\in V(C)$$ such that $$S_x(x)$$ is the interval $$[u,u'']$$. Choose some element $$x''\in X$$ such that there exists a path from $$x''$$ to $$u''$$ that does not cross an edge contained in *C*. Similarly, let $$v''\in V(C)$$ such that $$S_y(y)$$ is the interval $$[v'',v]$$. Choose some element $$y''\in X$$ such that there exists a path from $$y'$$ to $$v''$$ that does not cross an edge contained in *C*. Then since $$S'$$ is incompatible with $$S_x$$ and $$S_y$$ and displayed by *C*, it follows that $$S'(x'')= S'(y'')=S'(z)$$. Hence, $$S^*(z)\subseteq S'(z).$$ But then $$S^* $$ and $$S'$$ are not incompatible which is impossible. Thus $$S'$$ cannot be incompatible with both of $$S_x$$ and $$S_y$$, as claimed.

To see that $S^*$ is compatible with every split in $\Sigma$, we may, in view of the above claim, assume without loss of generality that $S'$ is compatible with $S_x$. Then Lemma 2 applied to $S'$, $S^*$, and $S_x$ implies $S_x(x) \subsetneq S'(x)$. We distinguish between the cases that ($\alpha$) $S_y$ and $S'$ are compatible and ($\beta$) that they are incompatible.

**Case** ($\alpha$) Since $S_y$ and $S'$ are compatible, similar arguments as above imply that $S_y(y) \subsetneq S'(y)$. Then the definition of $S^*$ combined with the assumption that $S'$ and $S^*$ are incompatible implies that $S'(x)\not =S'(y)$. But then $S'$ and $S_0$ must be compatible, and so, $S'(x)\subseteq S_0(x)$ or $S_0(x)\subseteq S'(x)$ must hold. If $S'(x)\subseteq S_0(x)$ held then $S'\in \Sigma _x$ which is impossible in view of the choice of $S_x$ as $S_x(x) \subsetneq S'(x)$. Thus, $S_0(x)\subseteq S'(x)$ must hold. But then $S_y(y)\subsetneq S'(y)\subseteq S_0(y)$ and so $S'\in \Sigma _y$ which is impossible in view of the choice of $S_y$. Thus, Case ($\beta$) must hold.

**Case** ($\beta$) Since $S_y$ and $S'$ are incompatible, the split

$$\begin{aligned} S''=S'(x)\cap \overline{S_y(y)}|\overline{S'(x)}\cup S_y(y) \end{aligned}$$

is contained in $\Sigma$ because $\Sigma$ is ${\mathcal {I}}$-intersection closed and clearly displayed by *C*. Note that $x\in \overline{S''(y)}$ and so $S''(x)=\overline{S''(y)}$ must hold. Moreover, since $S'$ and $S^*$ are incompatible, we cannot have $S''(x)=S_x(x)$ as $S_x$ and $S^*$ are compatible. But then $S_0$ and $S''$ cannot be compatible. Indeed, if $S_0$ and $S''$ were compatible then since $y\in S_0(y)\cap S''(y)$, $x\in \overline{S_0(y)}\cap \overline{S''(y)}$, and, because of $S_x(x)\subsetneq S'(x)$, also $\overline{S_0(y)}\cap S''(y)=S_0(x)\cap S''(y)=S_0(x)\cap (\overline{S'(x)}\cup S_y(y))\subseteq S_0(x)\cap \overline{S_x(x)}\not =\emptyset$ holds, it follows that $\overline{S''(y)}\subseteq \overline{S_0(y)}$, as required. Hence, $S''(x)= \overline{S''(y)}\subseteq \overline{S_0(y)}=S_0(x)$ and so $S''\in \Sigma _x$ which is impossible in view of the choice of $S_x$ as $S_x(x)\not =S''(x)$ and $S_0(x)\not =S''(x)$. Thus, $S_0$ and $S''$ must be incompatible. But this is also impossible since the interval on *C* corresponding to $S''(x)$ contains the interval [*x*, *z*] which induces the split $S_0$. Consequently, $S_0$ and $S''$ must be compatible. This final contradiction completes that proof that $S^*$ is compatible with every split in $\Sigma$.

To conclude, let *G* be a new graph obtained from *N* by adding a subdivision vertex *r* and $r'$, respectively, to each of two edges whose deletion induces the split $S^*$ (see Fig. 4b for an illustration). Then, the graph $G'$ obtained from *G* by identifying *r* and $r'$ is again a 1-nested network on *X*. By construction, $S_0 \in \hat{\Sigma }:=\{S \in \Sigma (N) : \text { S is incompatible with } S^*\}$ clearly holds and so $\Sigma (G')=\Sigma (N)-\hat{\Sigma } \subsetneq \Sigma (N)$. Since, by the above, every split in $\Sigma$ is compatible with $S^*$, it follows that $\Sigma \subseteq \Sigma (G')$. But this is impossible in view of the choice of *N*. Hence, the split $S_0$ cannot exist and, thus, $\Sigma =\Sigma (N)$.

(ii) Suppose *N* is a maximal partially resolved 1-nested network. Assume first that *N* is a level-1 network on *X* and, for contradiction, that there exists some split *S* of *X* not contained in $\Sigma (N)$ that is compatible with every split in $\Sigma (N)$. Then $S'$ cannot be a trivial split of *X*. Let $N'$ be the graph obtained from *N* by deleting from each cycle of *N* one of its edges and suppressing resulting degree two vertices. Clearly $N'$ is a phylogenetic tree on *X*. Since every non-leaf vertex of *N* has degree three, every such vertex in $N'$ must also have degree three. Hence, $\Sigma (N')$ is a maximal compatible split system on *X*. Since *S* is compatible with every split of $\Sigma (N)$ and $\Sigma (N')\subseteq \Sigma (N)$, it follows that $\Sigma (N')\cup \{S\}$ is also compatible which is impossible in view of the maximality of $\Sigma (N')$.

Conversely, assume that there exists no split of *X* not contained in $\Sigma (N)$ that is compatible with every split in $\Sigma (N)$. Then if *N* is not level-1, it contains a vertex *v* of degree $k \ge 4$, that does not belong to a cycle of *N*. Let $X_1,\ldots ,X_k$ be the partition of *X* obtained by deletion of *v* (suppressing incident edges). Then there exist $i,j\in \{1,\ldots , k\}$ distinct, say $i=1$ and $j=2$, such that the split $S:=X_1 \cup X_2| \bigcup _{i=3}^{k} X_i$ is compatible with every split in *N*. Since *S* does not belong to $\Sigma (N)$, this is impossible. $\square$

Note that Theorem 1(i) provides a way to decide for a split system $\Sigma$ if there exists a 1-nested network *N* such that $\Sigma =\Sigma (N)$ holds. However, it does not provide a tool for constructing such a network. The provision of such a tool is the purpose of the next two sections. Also note that in view of the relationship between 1-nested networks and *PC*-trees, Theorem 1(i) may be viewed as a consequence of Kleinman et al (2013), Proposition 4.7. Since the arguments used to establish Kleinman et al (2013), Proposition 4.7 are based on a relationship between so-called pre-pyramids and PQ-trees whereas the focus of our paper is on the development and study of a closure for split systems in a phylogenetic network context we prefer to present an independent proof of Theorem 1(i). In particular, this ensures that the paper is also self-contained. Furthermore, we remark that, due to the link between phylogenetic networks and cactus models described above, Theorem 1(i) may be viewed as the companion result for the algorithm presented in Brandes and Cornelsen (2010). That algorithm takes as input a split system $\Sigma$ and finds a cactus model $\Omega$ for $\Sigma$, if such a model exists. Note however that although the split system $\Sigma (\Omega )$ induced by $\Omega$ by taking (set inclusion) minimal cuts always contains $\Sigma$ it need not equal $\Sigma$.

## Optimality and the Analogue of the Splits Equivalence Theorem

As is easy to see, any circular split system on some set *X* can be represented in terms of a 1-nested network $N_{\Sigma }$ on *X* by first subdividing a cycle *C* by |*X*| vertices, then assigning the elements of *X* to the vertices of *C* according to their induced circular ordering, and, finally, attaching to each vertex *v* of *C* a pendant edge *e* and shifting the element of *X* labeling *v* to the degree one vertex of *e*. As the 1-nested network *N* depicted in Fig. 1b indicates for the split system $\Sigma$ comprising of all splits of the form $x|X-\{x\}$ where $x\in X:=\{1,\ldots ,8\}$ together with the splits 81|234567, 78|123456, 781|23456, 234|56781, 34|567812, 345|67812, 2345|6781, 3456|7812 and 56|78123, the network $N_{\Sigma }$ is generally not optimal. Put differently, $N_{\Sigma }$ displays a total of ${|X| \atopwithdelims ()2}$ distinct splits of *X* (including those in $\Sigma$) whereas the 1-nested network *N* depicted in that figure also displays all splits of $\Sigma$ and postulates fewer additional splits. Furthermore, the 1-nested network pictured in Fig. 2 also displays $\Sigma$ and so does the subgraph in terms of the bold edges of the Buneman graph $G(\Sigma )$ pictured in Fig. 1c where we suppress degree two vertices.

This and the next section are devoted to clarifying the above phenomenon. In particular, we show next that for any circular split system $\Sigma$ on *X* it is possible to construct a (in a well-defined sense) optimal 1-nested network on *X* in $O(n(n+|\Sigma |^2))$ time (Theorem 3). Central to our proof is Theorem 2 in which we characterize circular split systems whose $\mathcal I$-intersection closure is (set inclusion) maximal in terms of their so-called incompatibility graphs. As a consequence, we obtain as Corollary 2 the 1-nested analogue of the fundamental “splits equivalence theorem” for phylogenetic trees (see Sect. 1).

We start with introducing some more terminology. Suppose $\Sigma$ is a circular split system on *X*. Then we say that $\Sigma$ is *maximal circular* if for all circular split systems $\Sigma '$ on *X* that contain $\Sigma$, we have $\Sigma =\Sigma '$. As the next result illustrates, maximal circular split systems of *X* and 1-nested networks on *X* are closely related.

### Lemma 3

A split system $\Sigma$ on *X* is maximal circular if and only if there exists a simple level-1 network *N* on *X* such that $\Sigma = \Sigma (N)$.

### Proof

Let $\Sigma$ be a split system on *X*. Assume first that $\Sigma$ is maximal circular. Then there exists a simple level-1 network *N* on *X* such that $\Sigma \subseteq \Sigma (N)$. Since $\Sigma (N)$ is clearly a circular split system on *X* the maximality of $\Sigma$ implies $\Sigma = \Sigma (N)$.

Conversely, assume that *N* is a simple level-1 network such that $\Sigma = \Sigma (N)$. Then since $\Sigma (N)$ is a circular split system on *X* so is $\Sigma$. Assume for contradiction that $\Sigma$ is not maximal circular, that is, there exists a split $S=A|\bar{A}\in \Sigma$ that is not contained in $\Sigma (N)$. Then *A* and $\bar{A}$ are both intervals in the circular ordering of *X* induced by $\Sigma (N)$. Hence, *S* is induced by a minimal cut of *N*. Consequently, $S\in \Sigma (N)$ which is impossible. $\square$

Note that since a maximal circular split system on *X* must necessarily contain all 2-splits of *X* obtainable as a minimal cuts in the associated simple level-1 network on *X*, it follows that that ordering of *X* is unique. The next result suggests that systems of such splits suffice to generate a maximal circular split system. To state it, suppose $\sigma : x_1, \ldots , x_{n-1}, x_n, x_{n+1}:=x_1$ is a circular ordering of *X* and put $\Sigma _{\sigma }:=\{\{x_i,x_{i+1}\}| X-\{x_i,x_{i+1}\}\, :\, 1\le i\le n\}$. Clearly, $\Sigma _{\sigma }$ is a circular split system on *X*.

In view of Lemma 3, we say that a circular ordering *displays* a split system $\Sigma$ if $\Sigma$ is displayed by the simple level-1 network associated with $\Sigma$.

### Lemma 4

$\mathcal I(\Sigma _{\sigma })$ is a maximal circular split system on *X*, for any circular ordering $\sigma$ of *X*.

### Proof

Since the result is trivial for $n=3$, we may assume without loss of generality that $n \ge 4$. Let $\sigma : x_1, \ldots , x_{n-1}, x_n, x_{n+1}:=x_1$ be a circular ordering of *X*. We proceed by induction on the size $1 \le l \le \frac{n}{2}$ of a split *S* displayed by $\sigma$. Suppose first that $l=1$. Then there exists some $i\in \{1,\ldots , n\}$ such that $S=x_i|X-\{x_i\}$. Clearly, $S_1=\{x_i,x_{i-1}\}|X-\{x_i,x_{i-1}\}$ and $S_2=\{x_i,x_{i+1}\}|X-\{x_i,x_{i+1}\}$ are contained in $\Sigma _{\sigma }$ and incompatible. Hence, $S=S_1(x_i)\cap S_2(x_i)|X-(S_1(x_i)\cap S_2(x_i)) \in \iota (S_1,S_2)\subseteq {\mathcal {I}}(\Sigma _{\sigma })$.

Now assume that $l\ge 2$ and that all splits of *X* displayed by $\sigma$ of size at most $l-1$ are contained in ${\mathcal {I}}(\Sigma _{\sigma })$. Since *S* is displayed by $\sigma$, there exists some $i\in \{1,\ldots , n\}$ such that $S=[x_i, x_{i+l-1}]|X-[x_i, x_{i+l-1}]$. Without loss of generality, we may assume that $i=1$. Then $S=[x_1,x_l]|X-[x_1,x_l]$. Consider the splits $S_1=[x_1, x_{l-1}]|X-[x_1, x_{l-1}]$ and $S_2=\{x_{l-1},x_l\}|X-\{x_{l-1}, x_l\}$ displayed by $\sigma$. By induction, $S_1,S_2\in {\mathcal {I}}(\Sigma _{\sigma })$ since the size of $S_2$ is two and that of $S_1$ is at most $l-1$. Furthermore, $S_1$ and $S_2$ are incompatible. Since $S=S_1(x_{l-1})\cup S_2(x_{l-1})|X-(S_1(x_{l-1})\cup S_2(x_{l-1})) \in \iota (S_1,S_2)\subseteq {\mathcal {I}}(\Sigma _{\sigma })$, the lemma follows. $\square$

We next employ Lemma 4 to obtain a sufficient condition on a circular split system $\Sigma$ for its $\mathcal I$-intersection closure to be maximal circular. Central to this is the concept of the *incompatibility graph* $Incomp(\Sigma )$ associated with a split system $\Sigma$. The vertex set of that graph is $\Sigma$ and any two splits of $\Sigma$ are joined by an edge in $Incomp(\Sigma )$ if they are incompatible. We denote the set of connected components of $Incomp(\Sigma )$ by $\pi _0(\Sigma )$ and, by abuse of terminology, refer to the vertex set of an element in $\pi _0(\Sigma )$ as a *connected component of* $Incomp(\Sigma )$. For example, $Incomp(\Sigma _{\sigma })$ is a cycle of length $|\Sigma _{\sigma }|$ whenever $n\ge 5$. Furthermore, $\Sigma$ is compatible if and only if $|\Sigma _0|=1$ holds for all $\Sigma _0\in \pi _0(\Sigma )$.

We next clarify the relationship between the incompatibility graph and ${\mathcal {I}}$-intersection closure of a split system.

### Lemma 5

Suppose $\Sigma$ is a split system on *X*. Then for any two distinct connected components $\Sigma _1, \Sigma _2\in \pi _0(\Sigma )$ and any splits $S_1\in {\mathcal {I}}(\Sigma _1)$ and $S_2\in {\mathcal {I}}(\Sigma _2)$ we must have that $S_1$ and $S_2$ are compatible.

### Proof

Assume for contradiction that there exist two connected components $\Sigma _1,\Sigma _2\in \pi _0(\Sigma )$ and splits $S_1\in {\mathcal {I}}(\Sigma _1)$ and $S_2\in {\mathcal {I}}(\Sigma _2)$ such that $S_1$ and $S_2$ are incompatible. Then $S_1 \in \Sigma _1$ and $S_2 \in \Sigma _2$ cannot both hold as otherwise $\Sigma _1=\Sigma _2$. Assume without loss of generality that $S_1 \notin \Sigma _1$. Let $\Sigma ^0:=\Sigma _1 \subsetneq \Sigma ^1 \subsetneq \ldots \subsetneq \Sigma ^k:= {\mathcal {I}}(\Sigma _1)$, $k\ge 1$, be a finite sequence such that, for all $1\le i\le k$, a split in $\Sigma ^i$ either belongs to $\Sigma ^{i-1}$ or is an ${\mathcal {I}}$-intersection between two splits $S,S'\in \Sigma ^{i-1}$ and $\iota (S,S')\not \subseteq \Sigma ^{i-1}$. Then, there exists some $i^*>0$ such that $S_1 \in \Sigma ^{i^*} - \Sigma ^{i^*-1}$. After possibly renaming $S_1$, we may assume without loss of generality that $i^*$ is such that for all $1\le i\le i^*-1$ there exists no split in $\Sigma ^{i}$ that is incompatible with $S_2$. Hence, there must exist two splits *S* and $S'$ in $\Sigma ^{i^*-1}$ distinct such that $S_1 \in \iota (S,S')$. Since $S_2$ and $S_1$ are incompatible, it follows that $S_2$ is incompatible with one of *S* and $S'$, which is impossible by the choice of $i^*$. $\square$

Armed with this result, we next relate for a split system $\Sigma$ the sets $\pi _0({\mathcal {I}}(\Sigma ))$ and $\pi _0(\Sigma )$ in Lemma 6. In particular, we show that ${\mathcal {I}}(\Sigma )$ can be obtained as the intersection closure of the connected components of $Incomp(\Sigma )$. Also, the set of connected components of ${\mathcal {I}}(\Sigma )$ can be obtained as the connected components of the intersection closure of the connected components of $Icomp(\Sigma )$.

### Lemma 6

Suppose $\Sigma$ is a split system on *X*. Then the following hold

(i) $$\mathcal {I}(\Sigma )=\bigcup _{\Sigma _0 \in \pi _0(\Sigma )} {\mathcal {I}}(\Sigma _0)$$.
(ii) $\pi _0({\mathcal {I}}(\Sigma _0)) \subseteq \pi _0({\mathcal {I}}(\Sigma ))$, for all $\Sigma _0\in \pi _0(\Sigma )$. In particular,
  $$\begin{aligned} \pi _0({\mathcal {I}}(\Sigma ))=\bigcup _{\Sigma _0 \in \pi _0(\Sigma )} \pi _0({\mathcal {I}}(\Sigma _0)). \end{aligned}$$

### Proof

(i) Let $\Sigma _0\in \pi _0(\Sigma )$ and put ${\mathcal {A}}:=\bigcup _{\Sigma ' \in \pi _0(\Sigma )} {\mathcal {I}}(\Sigma ')$. Note that since $\Sigma =\bigcup _{\Sigma ' \in \pi _0(\Sigma )} \Sigma '$, we trivially have $\Sigma \subseteq {\mathcal {A}} \subseteq {\mathcal {I}}(\Sigma )$. To see that ${\mathcal {I}}(\Sigma )\subseteq {\mathcal {A}}$ note that Lemma 5 implies that any two incompatible splits in ${\mathcal {A}}$ must be contained in the same connected component of ${\mathcal {I}}(\Sigma )$ and so must be their ${\mathcal {I}}$-intersection. Hence, ${\mathcal {A}}$ is ${\mathcal {I}}$-intersection closed. Since $\Sigma \subseteq {\mathcal {A}}$ we also have ${\mathcal {I}}(\Sigma ) \subseteq {\mathcal {I}}( {\mathcal {A}}) = {\mathcal {A}}$. Thus ${\mathcal {A}}={\mathcal {I}} (\Sigma )$.
(ii) Suppose $\Sigma _0 \in \pi _0(\Sigma )$ and let ${\mathcal {A}}\in \pi _0({\mathcal {I}}(\Sigma _0))$. To establish that ${\mathcal {A}}\in \pi _0({\mathcal {I}}(\Sigma ))$ note that since $\mathcal A$ is connected in $Incomp({\mathcal {I}}(\Sigma _0))$ it also is connected in $Incomp({\mathcal {I}}(\Sigma ))$. Hence, it suffices to show that every split in ${\mathcal {A}}$ is compatible with every split in ${\mathcal {I}}(\Sigma )-{\mathcal {A}}$. Suppose $S_1\in \mathcal A$ and $S_2\in {\mathcal {I}}(\Sigma )-{\mathcal {A}}= ({\mathcal {I}}(\Sigma )-{\mathcal {I}}(\Sigma _0)) \cup ({\mathcal {I}}(\Sigma _0)- {\mathcal {A}})$. If $S_2\in {\mathcal {I}}(\Sigma _0)- {\mathcal {A}}$, then, by definition, $S_1$ and $S_2$ are compatible. So assume that $S_2\in {\mathcal {I}}(\Sigma )-{\mathcal {I}}(\Sigma _0)$. Then Lemma 6(i) implies that $S_2$ is compatible with every split in ${\mathcal {I}}(\Sigma _0)$ and, thus, with $S_1$ as ${\mathcal {PA}}\subseteq {\mathcal {I}}(\Sigma _0)$.

$$\square $$

To establish the next result which is central to Theorem 3, we require a further notation. Suppose $\Sigma$ is a split system on *X*. Then we denote by $\Sigma ^-$ the split system obtained from $\Sigma$ by deleting all trivial splits on *X*.

### Theorem 2

Let $\Sigma$ be a circular split system on *X*. Then ${\mathcal {I}}(\Sigma )$ is a maximal circular split system on *X* if and only if the following two conditions hold:

(i) for all $x, y \in X$ distinct, there exists some $S \in \Sigma ^-$ such that $S(x) \ne S(y)$,
(ii) $Incomp(\Sigma ^-)$ is connected.

Moreover, if (i) and (ii) hold then there exists an unique, up to isomorphism and partial resolution, simple 1-nested network *N* on *X* such that $\Sigma \subseteq \Sigma (N)$.

### Proof

Let $\sigma : x_1, \ldots , x_n, x_{n+1}:=x_1$ denote an underlying circular ordering of *X* for $\Sigma$. Assume first that (i) and (ii) hold. We first show that ${\mathcal {I}} (\Sigma ^-)$ is maximal circular. To this end, it suffices to show that $\Sigma _{\sigma }\subseteq {\mathcal {I}}(\Sigma ^-)$ since this implies that ${\mathcal {I}}(\Sigma _{\sigma }) \subseteq {\mathcal {I}}({\mathcal {I}}(\Sigma ^-)) \subseteq {\mathcal {I}}({\mathcal {I}}(\Sigma ))= {\mathcal {I}}(\Sigma )$. Combined with the fact that, in view of Lemma 4, ${\mathcal {I}}(\Sigma _{\sigma })$ is maximal circular, it follows that ${\mathcal {I}}(\Sigma _{\sigma })={\mathcal {I}}(\Sigma ^-)= {\mathcal {I}}(\Sigma )$. Hence, ${\mathcal {I}}(\Sigma )$ is maximal circular.

Assume for contradiction that there exists some $i\in \{1,\ldots , n\}$ such that the split $S^*=x_ix_{i+1}|x_{i+2},\ldots , x_{i-1}$ of $\Sigma _2$ is not contained in ${\mathcal {I}}( \Sigma )$. Then, by assumption, there exist two splits *S* and $S'$ in $\Sigma$ such that $S(x_i) \ne S(x_{i-1})$ and $S'(x_{i+1}) \ne S'(x_{i+2})$. Let $P_{SS'}$ denote a shortest path in $Incomp(\Sigma )$ joining *S* and $S'$. Without loss of generality, let *S* and $S'$ be such that the path $P_{SS'}$ is as short as possible. Let $S_0=S, S_1,\ldots , S_k=S'$ denote that path. The next lemma is central to the proof

### Lemma 7

For all $0 \le j \le k$, we have $S_j(x_i)=S_j(x_{i+1})$.

### Proof

First observe that $S_j(x_i) = S_j(x_{i-1})$ and $S_j(x_{i+1})= S_j(x_{i+2})$ must hold for all $0< j < k$. Indeed, if there existed some $j\in \{1,\ldots , k-1\}$ such that $S_j(x_i) \not = S_j(x_{i-1})$ then the path $S_j, S_{j+1},\ldots , S_k$ would be shorter than $P_{SS'}$, in contradiction to the choice of *S* and $S'$. Similar arguments also imply that $S_j(x_{i+1})= S_j(x_{i+2})$ holds for all $j\in \{1,\ldots , k-1\}$.

Assume for contradiction that there exists $0 \le j \le k$ such that $S_j(x_i) \ne S_j(x_{i+1})$. Without loss of generality, we may assume that, for all $0\le l\le j-1$, we have $S_l(x_i)=S_l(x_{i+1})$. Then since a trivial split cannot be incompatible with any other split on *X* we cannot have $j\in \{0,k\}$. Thus, the splits $S_{j-1}$ and $S_{j+1}$ must exist. Note that they cannot be incompatible, since otherwise the path from *S* to $S'$ obtained by deleting $S_j$ from $P_{SS'}$ is shorter than $P_{SS'}$ which is impossible. So $S_{j-1}$ and $S_{j+1}$ must be compatible. Clearly, $x_i\in S_{j+1}(x_i)\cap S_{j-1}(x_i)$. We next establish that $\overline{S_{j+1}(x_i)}\cap \overline{S_{j-1}(x_i)}=\emptyset$ cannot hold implying that either $S_{j+1}(x_i)\cap \overline{S_{j-1}(x_i)}=\emptyset$ or $\overline{S_{j+1}(x_i)}\cap S_{j-1}(x_i)=\emptyset$.

Indeed, let $q \in \{1, \ldots , n\}$ such that $S_j=x_{i+1} \ldots x_{q}|x_{q+1} \ldots x_{i}$. We claim that $x_q \in \overline{S_{j+1}(x_i)} \cap \overline{S_{j+1}(x_i)}$. Assume by contradiction that $x_q \in S_{j-1}(x_i)$ and that $i\le q$. Then $S_{j-1}(x_i)$ is an interval of *X* containing $\{x_i,x_q\}$. Hence, either $S_{j-1}(x_i) \supseteq [x_i, x_q] \supseteq S_j(x_{i+1})$ or $S_{j-1}(x_i) \supseteq [x_q, x_i] \supseteq S_j(x_{i})$. But both are impossible in view of the fact that $S_{j-1}$ and $S_j$ are incompatible.

Now assume that $S_{j+1}(x_i)\cap \overline{S_{j-1}(x_i)}=\emptyset$, that is, $S_{j+1}(x_i)\subseteq S_{j-1}(x_i)$. We postulate that then $S_{j+1}(x_i)\subseteq S_0(x_i)$ must hold which is impossible since $x_{i-1}\in S_{j+1}(x_i)$ and $S_0(x_i)\not =S_0(x_{i-1})$. Indeed, the choice of *S* and $S'$ implies that $S_{j+1}$ and $S_l$ must be compatible, for all $0\le l\le j-2$. By Lemma 2 applied to $S_{j-1}$, $S_{j-2}$, and $S_{j+1}$, it follows that $S_{j+1}(x_i)\subseteq S_{j-2}(x_i)$ or $S_{j-2}(x_i)\subseteq S_{j+1}(x_i)$. In the latter case, we obtain $S_{j-2}(x_i)\subseteq S_{j-1}(x_i)$ which is impossible since $S_{j-1}$ and $S_{j-2}$ are incompatible. Thus, $S_{j+1}(x_i)\subseteq S_{j-2}(x_i)$. Repeated application of this argument implies that, for all $0\le l\le j-2$, we have $S_{j+1}(x_i)\subseteq S_l(x_i)$, as required.

Finally, assume that $S_{j-1}(x_i)\cap \overline{S_{j+1}(x_i)}=\emptyset$, that is, $S_{j-1}(x_i)\subseteq S_{j+1}(x_i)$. Then similar arguments as in the previous case imply that $S_{j-1}(x_i)\subseteq S_k(x_i)$. But this is impossible since $x_{i+1}, x_{i+2} \in S_{j-1}(x_i)$ and $S_k(x_i)=S_k(x_{i+1})\not =S_k(x_{i+2})$. Thus, $S_j(x_i)=S_j(x_{i+1})$ must hold for all $0 \le j \le k$. This concludes the proof of Lemma 7. $\square$

Continuing with the proof of Theorem 2, we claim that the splits

$$\begin{aligned} T_j:=T_{j-1}(x_i) \cap S_j(x_i)| \overline{T_{j-1}(x_i)} \cup \overline{S_j(x_i)} \end{aligned}$$

where $$j\in \{1,\ldots ,k\}$$ and $$T_0:=S_0$$ are contained in $${\mathcal {I}}(\Sigma )$$. Assume for contradiction that there exists some $$j\in \{0,\ldots ,k\}$$ such that $$T_j\not \in {\mathcal {I}}(\Sigma )$$. Then $$j\not =0$$ because $$S\in {\mathcal {I}}(\Sigma )$$, and $$j \not = 1$$ since $$T_1\in \iota (S,S_1)$$ and $$S,S_1\in \Sigma $$. Without loss of generality, we may assume that *j* is such that for all $$1\le l\le j-1$$, we have $$T_l\in {\mathcal {I}}(\Sigma )$$. Then $$ T_{j-1}$$ and $$ S_j$$ cannot be incompatible and so $$T_{j-1}(x_i) \subseteq S_j(x_i)$$, or $${S_j(x_i)} \subseteq T_{j-1}(x_i)$$, or $$\overline{S_j(x_i)} \subseteq T_{j-1}(x_i)$$ must hold. But $$S_j(x_i) \subseteq T_{j-1}(x_i)$$ cannot hold since then $$\overline{S_{j-1}(x_i)}\subseteq \overline{T_{j-2}(x_i)} \cup \overline{S_{j-1}(x_i)} = \overline{T_{j-1}(x_i)}\subseteq \overline{S_j(x_i)}$$ which is impossible as $$S_{j-1}$$ and $$S_j$$ are incompatible. Also, $$\overline{S_j(x_i)} \subseteq T_{j-1}(x_i)$$ cannot hold since then $$\overline{S_j(x_i)} \subseteq T_{j-1}(x_i)=T_{j-2}(x_i)\cap S_{j-1}(x_i) \subseteq S_{j-1}(x_i)$$ which is again impossible as $$S_{j-1}$$ and $$S_j$$ are incompatible. Thus, $$T_{j-1}(x_i) \subseteq S_j(x_i)$$ and so $$T_j(x_i)=T_{j-1}(x_i)$$. Consequently, $$T_j = T_{j-1}\in {\mathcal {I}}(\Sigma )$$ which is also impossible and therefore proves the claim. Thus, $$T_j\in \mathcal {I}(\Sigma )$$, for all $$0\le j\le k$$. Combined with Lemma 7, it follows that, for all $$0 \le j \le k$$, we also have $$T_j(x_i)=T_j(x_{i+1})$$. Consequently, $$\{x_i,x_{i+1}\} \subseteq T_k(x_i)$$. Combined with the facts that $$T_k(x_i)$$ is an interval on *X* and $$x_{i-1} \notin S_0(x_i)$$, and similarly, $$x_{i+2} \notin S_k(x_i)$$ it follows that $$\{x_i,x_{i+1}\} = T_k(x_i)$$. Hence, $$S^*=T_k \in {\mathcal {I}}(\Sigma )$$, which is impossible. Thus, $$\Sigma _{\sigma }\subseteq {\mathcal {I}}( \Sigma ^-)$$ and so $${\mathcal {I}}(\Sigma _{\sigma }) ={\mathcal {I}}( \Sigma ^-)$$.

Conversely, assume that ${\mathcal {I}}(\Sigma )$ is maximal circular. Then ${\mathcal {I}}(\Sigma )$ clearly satisfies Properties (i) and (ii) that is, for all $x,y\in X$ distinct there exists some $S\in {\mathcal {I}}(\Sigma )^-$ such that $S(x)\not =S(y)$ and $Incomp({\mathcal {I}}(\Sigma )^-)$ is connected. We need to show that $\Sigma$ also satisfies Properties (i) and (ii). Assume for contradiction that $\Sigma$ does not satisfy Property (i). Then there exist $x,y\in X$ distinct such that for all splits $S\in \Sigma ^-$, we have $S(x)=S(y)$. Let $S\in {\mathcal {I}}(\Sigma )$ such that $S(x)\not =S(y)$ and let $S_1,S_2,\ldots ,S_l=S$ denote a sequence in ${\mathcal {I}}(\Sigma )$ such that $S_i\in \iota (S_{i-1},S_{i-2})$, for all $3\le i\le l$. Without loss of generality, we may assume that *l* is such that $S_i(x)=S_i(y)$, for all $3\le i\le l-1$. Then $S_j(x)=S_j(y)$, for all $j\in \{l-1,l-2\}$ and thus $S(x)=S(y)$ which is impossible.

Next, assume for contradiction that $\Sigma$ does not satisfy Property (ii). Let $\Sigma _1$ and $\Sigma _2$ denote two disjoint connected components of $Incomp(\Sigma ^-)$. For $i=1,2$, let ${\mathcal {A}}_i\in \pi _0({\mathcal {I}}(\Sigma _i)^-)$ such that $\Sigma _i\subseteq {\mathcal {A}}_i$. Then, $2\le |\Sigma _i|\le |{\mathcal {A}}_i|$, for all $i=1,2$. Combined with Lemma 6(ii), we obtain ${\mathcal {A}}_1,{\mathcal {A}}_2\in \pi _0({\mathcal {I}}(\Sigma )^-)$. Since $Incomp({\mathcal {I}}(\Sigma )^-)$ is connected, it follows for $i=1,2$ that ${\mathcal {A}}_i\subseteq {\mathcal {I}}(\Sigma _i)^-\subseteq {\mathcal {I}}(\Sigma )^- ={\mathcal {A}}_i$. Thus, ${\mathcal {I}}(\Sigma _1)^-={\mathcal {I}}(\Sigma ^-)={\mathcal {I}}(\Sigma _2)^-$ and so the incompatibility graphs $Incomp({\mathcal {I}}(\Sigma _1)^-)$, $Incomp({\mathcal {I}}(\Sigma )^-)$ and $Incomp({\mathcal {I}}(\Sigma _2)^-)$ all coincide. Suppose $S\in \Sigma _1$ and $S'\in \Sigma _2$ and let *P* denote a shortest path in $Incomp({\mathcal {I}}(\Sigma )^-)$ joining *S* and $S'$. Then there must exist incompatible splits *S* and $S'$ in *P* such that $S\in \Sigma _1\subseteq \mathcal I(\Sigma _1)^-$ and $S'\in {\mathcal {I}}(\Sigma _1)^-={\mathcal {I}}(\Sigma _2)^-$ which is impossible in view of Lemma 5.

The remainder of the theorem follows from the facts that, by Lemma 4, ${\mathcal {I}}(\Sigma )$ is maximal circular that, by Lemma 3, there exists a simple level-1 network *N* such that ${\mathcal {I}}(\Sigma )=\Sigma (N)$, that, by Corollary 1(ii), a 1-nested network displays ${\mathcal {I}}(\Sigma )$ if and only if it displays $\Sigma$, and that the split system $\Sigma _{\sigma }$ uniquely determines the underlying circular ordering of *X*.

Armed with this characterization, we are now ready to establish Theorem 3.

### Theorem 3

Given a circular split system $\Sigma$ on *X*, it is possible to build, in time $O(n(n+|\Sigma |^2))$, a 1-nested network *N* on *X* such that $\Sigma \subseteq \Sigma (N)$ holds and $|\Sigma (N)|$ is minimal. Furthermore, *N* is unique up to isomorphism and partial resolution.

### Proof

Suppose $\Sigma$ is a circular split system on *X*. Put $\{V_1,\ldots ,V_l\}=\pi _0(\Sigma )$. Without loss of generality, we may assume that there exists some $j\in \{1,\ldots ,l\}$ such that $|V_i|=1$ holds for all $1\le i\le j-1$ and $|V_i|\ge 2$ for all $j\le i\le l$. Since $Incomp(\Sigma )$ has $l-j+1$ connected components with at least two vertices there exist $l-j+1$ simple 1-nested networks $N_i$ such that $V_i\subseteq \Sigma (C_i)$ holds for the unique cycle $C_i$ of $N_i$. By Theorem 2, it follows for all $j\le i\le l$ that $\Sigma (C_i)={\mathcal {I}}(V_i)$ and that $Q_i \subseteq \mathcal I(V_i)$, where $Q_i$ denotes the set of m-splits of $C_i$.

We claim that the split system $\Sigma '$ on *X* given by

$$\begin{aligned} \Sigma '=\bigcup _{i=1}^{j-1}V_i\cup \bigcup _{i=j}^lQ_i\cup \bigcup _{x\in X}\{x|X-x\} \end{aligned}$$

is compatible. Since $\Sigma$ is circular, there exists a 1-nested network *N* on *X* such that $\Sigma \subseteq \Sigma (N)$. Without loss of generality, we may assume that *N* is such that $|\Sigma (N)|$ is minimal among such networks. For clarity of exposition, we may furthermore assume that *N* is maximal partially resolved. Then for all $j\le i\le l$ there exists a cycle $Z_i$ in *N* such that $V_i\subseteq \Sigma (Z_i)$. In fact, ${\mathcal {I}}(V_i)= \Sigma (Z_i)$ must hold for all such *i*. Combined with the minimality of $\Sigma (N)$, it follows that there exists a one-to-one correspondence between the cycles of *N* and the set ${\mathcal {A}}:={\mathcal {I}}(V_i)\,:\, \{j\le i\le l\}$ that maps a cycle *C* of *N* to the split system $\Sigma _C\in {\mathcal {A}}$ such that for some $i^*\in \{j,\ldots ,l\}$ we have $\Sigma _C={\mathcal {I}}(V_{i^*})$ and $V_{i^*}\subseteq \Sigma (C)$. Furthermore, for all $1\le i\le j-1$ there exists a cut edge $e_i$ of *N* such that the split $S_{e_i}$ induced on *X* by deleting $e_i$ is the unique element in $V_i$.

Let *T*(*N*) denote the phylogenetic tree on *X* obtained from *N* by first shrinking every cycle *Z* of *N* to a vertex $v_Z$ and then suppressing all resulting degree two vertices. Since this operation clearly preserves the splits in $Q_i$, $j\le i\le l$, and also does not affect the cut edges of *N* (in the sense that a cut edge of *T*(*N*) might correspond to a path in *N* of length at most 3 involving a cut edge of *N* and one or two m-splits), it follows that $\Sigma ' =\Sigma (T(N))$. Since any split system displayed by a phylogenetic tree is compatible, the claim follows.

Since, in addition, $\Sigma '$ also contains all trivial splits on *X*, it follows by the “splits equivalence theorem” (see Sect. 1) that there exists a unique (up to isomorphism) phylogenetic tree *T* on *X* such $\Sigma (T)=\Sigma '$. Hence, *T*(*N*) and *T* must be isomorphic. But then reversing the aforementioned cycle-shrinking operation that gave rise to *T*(*N*) results in a 1-nested network $N'$ on *X* such that $\Sigma (N)=\Sigma (N')$. Consequently, $N'$ and *N* are isomorphic and so $\Sigma \subseteq \Sigma (N')$. Note that similar arguments also imply that *N* is unique up to partial resolution and isomorphism.

To see the remainder of the theorem, note first that finding $Incomp(\Sigma )$ can be accomplished in $O(n|\Sigma |^2)$ time. Combined with the facts that *X* has at most *n* cycles and any binary unrooted phylogenetic tree on *X* has $2n-3$ cut edges it follows that $N'$ can be constructed in $O(n^2+n|\Sigma |^2)$ time. $\square$

In consequence of Theorems 1 and 3, we obtain the 1-nested analogue of the “splits equivalence theorem” for phylogenetic trees (see Sect. 1).

### Corollary 2

Suppose $\Sigma$ is a split system on *X* that contains all trivial splits of *X*. Then there exists a 1-nested network *N* on *X* such that $\Sigma =\Sigma (N)$ if and only if $\Sigma$ is circular and ${\mathcal {I}}$-intersection closed. Moreover, if such a network *N* exists, then it is unique up to isomorphism and partial resolution and can be constructed in $O(n(n+|\Sigma |^2))$ time.

As observed in Sect. 2, a 1-nested network also induces a multi-set of splits. This raises the question of an 1-nested analogue of the “splits equivalence theorem” (see Sect. 1) for such collections. We will settle this question elsewhere.

## Optimality and the Buneman Graph

In this section, we investigate the interplay between the Buneman graph $G(\Sigma )$ associated with a circular split system $\Sigma$ and a 1-nested network displaying $\Sigma$. More precisely, we first associate with a circular split system $\Sigma$ a certain subgraph of $G(\Sigma )$ which we obtain by replacing each block of $G(\Sigma )$ by a structurally simpler graph which we call a marguerite. As it turns out, marguerites hold the key for constructing optimal 1-nested networks from circular split systems.

### Marguerites and Blocks

In this section, we first focus on the Buneman graph of a maximal circular split system and then introduce and study the novel concept of a marguerite. We start with collecting some relevant results.

For $\Sigma$ a split system on *X*, the following five properties of $G(\Sigma )$ are well known (see, e.g., Dress et al. (2012), Chapter 4).

(Bi) The split system $\Sigma (G(\Sigma ))$ Bu-displayed by $G(\Sigma )$ is $\Sigma$.
(Bii) For $\phi \in V(\Sigma )$ let ${\mathrm {\min }}(\phi (\Sigma ))$ denote the set inclusion minimal elements in $\phi (\Sigma ):=\{\phi (S)\,:\, S\in \Sigma \}$ and let $\Sigma ^{(\phi )}$ denote the set of pre-images of the elements in ${\mathrm {\min }}(\phi (\Sigma ))$ under $\phi$. Then a vertex $\psi \in V(\Sigma )$ is adjacent with $\phi$ if and only if there exists some split $S^*\in \Sigma ^{(\phi )}$ such that $\psi (S^*)=\overline{\phi (S^*)}$ and $\psi (S)=\phi (S)$, otherwise. In particular, $|\Sigma ^{(\phi )}|$ is the degree of $\phi$ in $G(\Sigma )$.
(Biii) In case $\Sigma$ contains all trivial splits on *X*, then $\Sigma$ is compatible if and only if, when identifying each Kuratowski map $\phi _x$ with its underlying element $x\in X$, $G(\Sigma )$ is a unrooted phylogenetic tree on *X* for which $\Sigma (G(\Sigma ))=\Sigma$ holds. Moreover, and up to isomorphism, $G(\Sigma )$ is unique.

Note that for any two distinct compatible splits *S* and $S'$ of *X* there must exist a unique subset $A \in S\cup S'$, say $A\in S$, such that $A \cap A'\not = \emptyset$ holds for all $A'\in S'$. Denoting that subset by $\max (S|S')$, we obtain

(Biv) For $\Sigma ',\Sigma ''\in \pi _0(\Sigma )$ distinct we have $\max (S'|S'')= \max (S'|S''')$, for all $S'\in \Sigma '$ and all $S'',S'''\in \Sigma ''$. In consequence, $\max (S'|\Sigma ''):=\max (S'|S'')$ is well defined where $S'\in \Sigma '$ and $S''\in \Sigma ''$ (Dress et al. 2012, Section 5).
(Bv) The blocks of $G(\Sigma )$ are in 1–1 correspondence with the connected components of $Incomp(\Sigma )$. More precisely, the map $\Theta : \pi _0(\Sigma )\rightarrow \mathfrak {Bl}(G(\Sigma ))$: $\Sigma _0\mapsto B(\Sigma _0):=\{\phi \in V(\Sigma ): \phi (S)=\max (S|\Sigma _0) \text{ holds } \text{ for } \text{ all } S\in \Sigma -\Sigma _0\}$ is a bijection (Dress et al. 2011, Theorem 5.1) where $\mathfrak {Bl}(G(\Sigma ))$ denotes the set of blocks of $G(\Sigma )$.

To illustrate these definitions, consider again the Buneman graph depicted in Fig. 1c and the splits $S=78|1\ldots 6$ and $S'=18|2\ldots 7$ both of which are Bu-displayed by that graph. Then for the marked vertex $\phi$, we have $\phi (S)=\{7,8\}$. The block marked $\mathcal B_1$ in that figure corresponds via $\Theta$ to the connected component $\Sigma _0=\{S,S'\}$ and $\max (S'|\Sigma _0)=X-\{2,3,4\}$.

For the following, assume that $k\ge 4$ and that $Y=\{X_1,\ldots , X_k\}$ is a partition of *X*. For clarity of exposition, also assume that $|X_i|=1$, for all $1\le i\le k$ and that the unique element in $X_i$ is denoted by *i*. Further, assume that $\sigma$ is the lexicographical ordering of *X* where we put $k+1:=1$. Let $\Sigma _k$ denote the maximal circular split system displayed by $\sigma$ bar the trivial splits of *X*. Since $\Sigma _k$ contains all 2-splits displayed by $\sigma$, it follows that $|\pi _0(\Sigma _k)|=1$. Hence, $G(\Sigma _k)$ is a block in view of Property (Bv). To better understand the structure of $G(\Sigma _k)$ consider for all $1 \le i \le k$ and for all $0 \le j < k-3$ the map:

$$\begin{aligned} \begin{array}{r c l} \phi _i^j : \Sigma _k \rightarrow \mathcal {P}(X):\,\,\,\, S \mapsto \left\{ \begin{array}{r l} \overline{S(i)} &{}\quad \text {if } S(i) \subseteq [i-j, i] \\ S(i) &{}\quad \text {otherwise.} \end{array} \right. \end{array} \end{aligned}$$

**Fig. 5:**
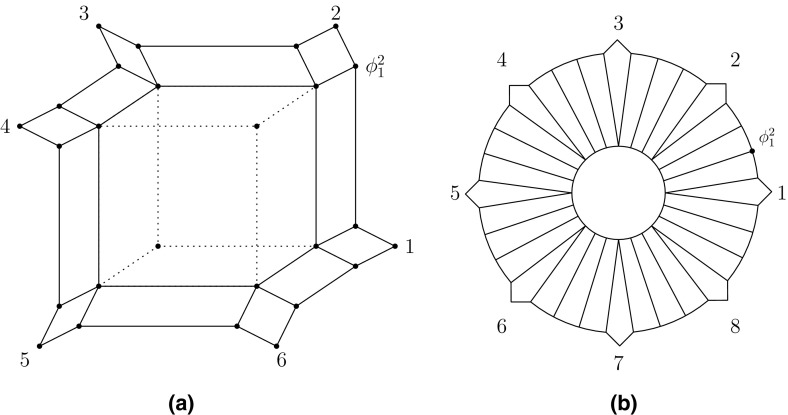
For $k=6$, we depict in **a** the Buneman graph $G(\Sigma _6)$ in terms of *bold* and *dashed edges* and the associated 6-marguerite $M(\Sigma _6)$ in terms of *bold edges*. In addition, we indicated the vertex $\phi _1^2$ of $G(\Sigma _6)$. We picture the 8-marguerite in **b** and indicate again the vertex $\phi _1^2$

For example, for $k=6$ and $k=8$ the map $\phi _1^2$ is indicated by a vertex in Fig. 5a, b, respectively.

To establish the next result, we associated with every element $i\in X$ the split system $\Sigma (i)^+:=\{S\in \Sigma _k\,:\, S(i+1)=S(i)\not =S(i-1)\}$. Then the partial ordering “$\preceq _i$” defined, for all $S,S'\in \Sigma _k$, by putting $S\preceq _i S'$ if $|S(i)|\le |S'(i)|$, is clearly a total ordering of $\Sigma (i)^+$ with minimal element $S^+_i=[i,i+1]|X-[i,i+1]$

#### Lemma 8

For any $k\ge 4$ the following statements hold:

(i) For all $i\in \{1\ldots ,k\}$ and all $0 \le j < k-3$ the map $\phi _i^j$ is a vertex of $G(\Sigma _k)$, $\phi _i^{k-3}=\phi _{i+1}^0$ holds, and $\Delta (\phi _i^j,\phi _i^{j+1})=\{[i-j-1,i]|X-[i-j-1,i]\}$. In particular, $\{\phi _i^j,\phi _i^{j+1}\}$ is an edge in $G(\Sigma _k)$.
(ii) For all $i\in \{1\ldots ,k\}$ and all $1\le j<k-3$, the map
  $$\begin{aligned} \begin{array}{r c l} \psi _i^j : \Sigma _k \rightarrow \mathcal {P}(X):\,\,\,\, S \mapsto \left\{ \begin{array}{r l} \overline{\phi _i^j(S)} &{}\quad \text {if } S=S^+_i \\ \phi _i^j(S) &{}\quad \text {otherwise.} \end{array} \right. \end{array} \end{aligned}$$
  is a vertex in $G(\Sigma _k)$ that is adjacent with $\phi _i^j$. Moreover, $\psi _i^{k-3}=\psi _{i+1}^0$ and $\{\psi _i^j,\psi _i^{j+1}\}$ is an edge in $G(\Sigma _k)$.

#### Proof

(i) Suppose $i\in \{1\ldots ,k\}$ and $0 \le j < k-3$. To see that $\phi _i^j\in V(\Sigma _k)$, we distinguish between the cases that (a) $j=0$, (b) $j=k-3$, and (c) $1\le j\le k-4$. Let $i\in \{1,\ldots , k\}$. Assume first that (a) holds and let $S\in \Sigma _k$. Then $\phi _i^0(S)=S(i)$ must hold since $\Sigma _k$ does not contain trivial splits. Moreover, $\phi _i^0(S)=\overline{S(i)}$ holds if and only if $S(i) \subseteq \{i\}$ if and only if *S* is the trivial split $i|X-i$. Thus, $\phi _i^0$ is a vertex in $G(\Sigma _k)$ in this case. Assume next that (b) holds. We claim that $\phi _i^{k-3}=\phi _{i+1}^0$. Assume again that $S\in \Sigma _k$. Observe that since $i-(k-3)\equiv i+3 ( \mod k)$ we have $S(i) \subseteq \{i-(k-3), \ldots , i\}$ if and only if $\{i+1,i+2\} \subseteq \overline{S(i)}$. We distinguish between the cases that ($\alpha$) $S(i)=S(i+1)$ and ($\beta$) $S(i)\not =S(i+1)$. Assume first that Case ($\alpha$) holds, that is, $S(i)=S(i+1)$. Then $\{i+1,i+2\} \not \subseteq \overline{S(i)}$. Combined with the observation made at the beginning of the proof of this case, we obtain $S(i)\not \subseteq \{i-(k-3), \ldots , i\}$ and, so, $\phi _i^{k-3}(S)=S(i)=S(i+1)=\phi _{i+1}^0(S)$. Next, assume that Case ($\beta$) holds, that is, $S(i)\not =S(i+1)$. Then $i+1\in \overline{S(i)}$. Since *S* cannot be a trivial split, it follows that $i+2\in \overline{S(i)}$ must hold too. Combined again with the observation made at the beginning of the proof of this case, it follows that $S(i) \subseteq \{i-(k-3), \ldots , i\}$. Thus, $\phi _i^{k-3}(S)=\overline{S(i)}=S(i+1)=\phi _{i+1}^0(S)$ which completes the proof of the claim. In combination with Case ($\alpha$), $\phi _i^{k-3}\in G(\Sigma _k)$ follows. So assume that (c) holds. Combining (a) with Property (Bii) and the fact that $\phi _i^0(S)=\phi _i^1(S)$ for all $S\in \Sigma _k-\{S^+_i \}$ and $\phi _i^0(S^+_i)=\overline{\phi _i^1(S^+_i )}$, it follows that $\phi _i^1$ is a vertex of $G(\Sigma _k)$. Similar arguments imply that if $\phi _i^l$ is a vertex in $G(\Sigma _k)$ then so is $\phi _i^{l+1}$. This concludes the proof of Case (c). That $\Delta (\phi _i^j,\phi _i^{j+1})=\{[i-j-1,i]|X-[i-j-1,i]\}$ holds for all $i\in \{1\ldots ,k\}$ and $0 \le j < k-3$ is an immediate consequence of the construction.
(ii) Suppose $i\in \{1\ldots ,k\}$ and $1\le j<k-3$. Then $\psi _i^j$ must be a vertex of $G(\Sigma _k)$ that is adjacent with $\phi _i^j$ in view of Property (Bii) as $S^+_i \in \Sigma ^{\phi _i^j}$. That $\psi _i^1=\psi _{i-1}^{k-3}$ holds is implied by the fact that the two splits in which $\psi _i^{k-3}$ and $\psi _{i+1}^1$ differ from $\phi _{i+1}^0$ are incompatible. That $\{\psi _i^j,\psi _i^{j+1}\}$ is an edge in $G(\Sigma _k)$ follows from the fact that $\{\phi _i^j,\phi _i^{j+1}\}$ is an edge in $G(\Sigma _k)$. $\square$

Bearing in mind Lemma 8, we next associate with $G(\Sigma _k)$ the *k-marguerite* $M(\Sigma _k)$ on *X*, that is, the subgraph of $G(\Sigma _k)$ induced by the set of maps $\phi _i^j$ and $\psi _i^l$ where $1\le i\le k$, $0\le j<k-3$ and $1\le l<k-3$. We illustrate this definition for $k=6,8$ in Fig. 5. Note that if *k* or *X* are of no relevance to the discussion then we shall simply refer to a *k*-marguerite on *X* as a *marguerite*.

Clearly, $G(\Sigma _k)$ and $M(\Sigma _k)$ coincide for $k=4,5$. To be able to shed light into the structure of *k*-marguerites for $k\ge 6$, we require some more terminology. Suppose $k\ge 4$ and $i\in \{0,\ldots , k\}$. Then we call a vertex of $M(\Sigma _k)$ of the from $\phi _i^0$ an *external vertex*. Moreover, we call for all $0\le j<k-3$ an edge of $M(\Sigma _k)$ of the form $\{\phi _i^j,\phi _i^{j+1}\}$ an *external edge*. Note that since $M(\Sigma _k)$ is in particular a subgraph of the $|\Sigma _k|$-dimensional hypercube, any split in $\Sigma _k$ not of the form $i,i+1| X-\{i,i+1\}$ is Bu-displayed in terms of four parallel edges of $M(\Sigma _k)$ exactly two of which are external.

### Gates

In this section, we establish that any partially resolved 1-nested network *N* can be embedded into the Buneman graph associated with $\Sigma (N)$. This allows us to bring to bear to such networks a wealth of results for the Buneman graph. Of particular interest to us are gated subsets of $V(\Sigma )$. A subset $Y\subseteq Z$ of a (proper) metric space (*Z*, *D*) is called a *gated* subset of *Z* if there exists for every $z\in Z$ a (necessarily unique) element $y_z\in Y$ such that $D(y,z)=D(y,y_z)+D(y_z,z)$ holds for all $y\in Y$. We refer to $y_z$ as the *gate* for *z* in *Y*.

We start with associating a metric space to the Buneman graph of a split system. Suppose $\Sigma$ is a split system on *X* such that for all *x* and *y* in *X* distinct there exists some $S\in \Sigma$ such that $S(x)\not =S(y)$. Then the map $D:V(\Sigma )\times V(\Sigma )\rightarrow {\mathbb {R}}_{\ge 0}: (\phi ,\phi ')\mapsto |\Delta (\phi ,\phi ')|$ is a (proper) metric on $V(\Sigma )$ (see, e.g., Dress et al. 2012, page 52) that is, *D* attains 0 only on the main diagonal, is symmetric, and satisfies the triangle inequality.

For $\Sigma$ a split system on *X* and $\Sigma '\in \pi _0(\Sigma )$, the following two additional properties of the Buneman graph, from Dress et al. (2011), turn out to be useful.

(Bvi) The map
  $$\begin{aligned} \begin{array}{r c l} V(\Sigma ') \rightarrow V(\Sigma ): \,\,\,\, \phi \mapsto (\begin{array}{r c l} \tilde{\phi } : \Sigma \rightarrow {\mathcal {P}}(X): \,\,\,\, S \mapsto \left\{ \begin{array}{r l} \phi (S) &{}\quad \text { if } S \in \Sigma ', \\ \mathrm {max}(S|\Sigma ') &{}\quad \text { otherwise} \end{array} \right. \end{array} \end{array} \end{aligned}$$
  is an isometry between $G(\Sigma ')$ and the block $B(\Sigma ')$ of $G(\Sigma )$.
(Bvii) For every map $\phi \in V(\Sigma )$, the map $\phi _{\Sigma '}$ given by
  $$\begin{aligned} \phi _{\Sigma '} : \Sigma (N) \rightarrow {\mathcal {P}}(X): \,\,\,\, S \mapsto \left\{ \begin{array}{r l} \phi (S) &{}\quad \text { if } S \in \Sigma ' \\ \mathrm {max}(S|\Sigma ') &{}\quad \text { otherwise,} \end{array} \right. \end{aligned}$$

is the gate for $\phi$ in $B(\Sigma ')$. We denote by $Gates(G(\Sigma ))$ the set of all vertices $\phi$ of $G(\Sigma )$ for which there exists a block $B\in \mathfrak {Bl}(G(\Sigma ))$ such that $\phi$ is the gate for some $x\in X$ in *B*.

#### Lemma 9

Suppose *N* is a 1-nested network on *X*. Then a block of $G(\Sigma (N))$ is either a cut edge or contains precisely one marguerite. Moreover, the gates of a marguerite *M* in $G(\Sigma (N))$ are the maps $\tilde{\phi }$ where $\phi$ is an external vertex of *M*.

#### Proof

Suppose $\Sigma '\in \pi _0(\Sigma (N))$. Note that $|\Sigma '|=1$ if and only if $B(\Sigma ')$ is a cut edge of $G(\Sigma (N))$. So assume that $|\Sigma '|\ge 2$. Then $B(\Sigma ')$ is a block of $G(\Sigma (N))$ and, so, there exists a unique cycle *C* of *N* of length $k\ge 4$ such that $\Sigma (C)=\Sigma '$. Let *Y* denote the partition of *X* induced by deleting all edges of *C* and let $\Sigma '_Y$ denote the split system on *Y* induced by $\Sigma (C)$. Then $\Sigma '_Y$ is of the form $\Sigma _k$. Hence, $G(\Sigma _Y')$ contains the *k*-marguerite $M(\Sigma '_Y)$. Combined with Property (Bvi), it follows that $G(\Sigma (N))$ contains the marguerite $M(\Sigma '_Y)$ (or, more precisely, the graph obtained by replacing for every external vertex $\phi _i^0$, $1\le i\le k$, the label $Y_i\in Y$ by the elements in $Y_i$).

To see the remainder of the lemma, suppose that *M* is a marguerite and assume that $k\ge 4$ such that $M=M(\Sigma _k)$. Let $Y=\{X_1,\ldots ,X_k\}$ denote the partition of *X* induced by $\Sigma _k$ and assume that $x\in X$. Then there must exist some $i\in \{1,\ldots ,k\}$ such that $x\in X_i$. Since $\phi _i^0$ is clearly the map

$$\begin{aligned} \begin{array}{r c l} \phi _i^0 : \Sigma _k \rightarrow \mathcal {P}(X):\,\,\,\, S=A|B \mapsto \left\{ \begin{array}{r l} A &{}\quad \text {if } X_i \subseteq A \\ B &{}\quad \text {if } X_i \subseteq B, \end{array} \right. \end{array} \end{aligned}$$

Properties (Bvi) and (Bvii) imply that $\tilde{ \phi _i^0}$ is the gate for *x* in *M*. $\square$

To be able to establish that any 1-nested partially resolved network *N* can be embedded as a (not necessarily induced) subgraph into the Buneman graph $G(\Sigma (N))$ associated with $\Sigma (N)$, we require again more terminology. Suppose *N* is a partially resolved 1-nested network and *v* is a non-leaf vertex of *N*. Then *v* is either incident with three or more cut edges of *N*, or there exists a cycle $C_v$ of *N* that contains *v* in its vertex set. In the former case, we choose one of them and denote it by $e_v$. In addition, we denote by $x_v\in X$ an element such that $e_v$ is not contained in any path in *N* from $x_v$ to *v*. In the latter case, we define $x_v$ to be an element in *X* such that no edge of $C_v$ is contained in any path in *N* from *v* to $x_v$.

#### Theorem 4

Suppose *N* is a 1-nested partially resolved network on *X*. Then the map $\xi :V(N)-X\rightarrow Gates(G(\Sigma (N)))$ defined by mapping every non-leaf vertex $v\in V(N)$ to the map

$$\begin{aligned} \xi (v):\Sigma (N)\rightarrow {\mathcal {P}}(X):\,\,\,\, S\mapsto \left\{ \begin{array}{r l} \max (S|\Sigma ^*) &{}\quad \text {if } S\in \Sigma (N)-\Sigma ^* \\ S(x_v) &{}\quad \text {else } \end{array} \right. \end{aligned}$$

is a bijection between the set of non-leaf vertices of *N* and the gates of $G(\Sigma (N))$ where $\Sigma ^*=\{S_{e_v}\}$ if *v* is contained in three or more cut edges of *N* and $\Sigma ^*=\Sigma (C_v)^-$ else. In particular, $\xi$ induces an embedding of *N* into $G(\Sigma (N))$ by mapping each leaf *x* of *N* to the leaf $\phi _x$ of $G(\Sigma (N))$ and replacing for any two adjacent vertices *v* and *w* of a cycle *C* of *N* of length *k* the edge $\{v,w\}$ by the path $\phi _i^0:=\xi (v) ,\phi _i^1,\ldots , \phi _i^{k-3}:=\xi (w)$.

#### Proof

Suppose *N* is a 1-nested network and put $\Sigma =\Sigma (N)$. To see that $\xi$ is well defined suppose $v\in V(N)-X$. Then *v* is either contained in three or more cut edges of *N* or *v* is a vertex of some cycle *C* of *N*. In the former case, we obtain $\{S_{e_v}\}\in \pi _0(\Sigma (N))$, and in the later, we have $C=C_v$ and $\Sigma (C_v)^-\in \pi _0(\Sigma )$. In either case, the definition of the element $x_v$ combined with Property (Bvii) implies $\xi (v)\in Gates(G(\Sigma ))$.

To see that $\xi$ is injective suppose *v* and *w* are two non-leaf vertices of *N* such that $\xi (v)=\xi (w)$. Assume for contradiction that $v\not =w$. It suffices to distinguish between the cases that (i) *v* and *w* are contained in the same cycle and that (ii) there exists a cut edge $e'$ on any path from *v* to *w*.

To see that (i) cannot hold, suppose that *v* and *w* are vertices on a cycle *C* of *N*. Then, $S(x_v) = \max (S|\Sigma (C)^-)=S(x_w)$ must hold for the m-split *S* obtained by deleting the two edges of *C* adjacent to *v* which is impossible. Thus (ii) must hold. Hence, there must exist a cut edge $e'$ on the path from *v* to *w*. Then $\xi (v)(S_{e'}) \ne \xi (w)(S_{e'})$ follows which is again impossible. Thus, $\xi$ must be injective.

To see that $\xi$ is surjective suppose $g\in Gates(G(\Sigma ))$. Then there exists some $x_g\in X$ and some block $B\in \mathfrak {Bl}(G(\Sigma ))$ such that *g* is the gate for $x_g$ in *B*. Let $\Sigma _B\in \pi _0(\Sigma (N))$ denote the connected component that, in view of Property (Bv), is in one-to-one correspondence with *B*. If there exists a cycle *C* of *N* such that $\Sigma (C)^-=\Sigma _B$, then let $v_g$ be a vertex of *N* such that no edge on any path from $v_g$ to $x_g$ crosses an edge of *C*. Then, by construction, $\xi (v_g)=g$. Similar arguments show that $\xi (v_g)=g$ must hold if $\Sigma _B$ contains precisely one split and thus corresponds to a cut edge of *N*. Hence, $\xi$ is also surjective and thus bijective.

The remainder of the theorem is straightforward. $\square$

Theorem 4 implies that by carrying out the two steps (Ci) and (Cii) stated in Corollary 3 any 1-nested partially resolved network *N* induces a 1-nested network $N(\Sigma (N))$ such that the split system $\Sigma (N(\Sigma (N)))$ induced by $N(\Sigma (N))$ is the split system $\Sigma (N)$ induced by *N*.

#### Corollary 3

Let $\Sigma$ be a split system on *X* for which there exists a 1-nested network *N* such that $\Sigma =\Sigma (N)$. Then $N(\Sigma )$ can be obtained from $G(\Sigma )$ by carrying out the following steps:

*(Ci)*: For all $x\in X$, replace each leaf $\phi _x$ of $G(\Sigma )$ by *x*, and
*(Cii)*: For all blocks *B* of $G(\Sigma )$ that contain a *k*-marguerite *M* for some $k\ge 4$, first add the edges $\{\phi _i^0,\phi _{i+1}^0\}$ for all $i \in \{1, \ldots , k\}$ where $k+1:=1$ and then delete all edges and all vertices of *B* not of the form $\phi _i^0$ for some $1\le i \le k$.

We next show that even if the circular split system under consideration does not satisfy the assumptions of Corollary 3, steps (Ci) and (Cii) still give rise to a, in a well-defined sense, optimal 1-nested network.

#### Theorem 5

Let $\Sigma$ be a circular split system on *X* that contains all trivial splits on *X*. Then $N:=N(\Sigma )$ is a 1-nested network such that:

(i) $$\Sigma \subseteq \Sigma (N)$$,
(ii) $|\Sigma (N)|$ is minimal among the 1-nested network satisfying *(i)*,
(iii) A vertex *v* of a cycle *C* of *N* is partially resolved if and only if the splits displayed by the edges of *C* incident with *v* belong to $\Sigma$.

Moreover, *N* is unique up to isomorphism and partial resolution.

#### Proof

(i) and (ii): Suppose for contradiction that there exists a 1-nested network $N'$ such that $\Sigma \subseteq \Sigma (N')$ and $|\Sigma (N')|< |\Sigma (N(\Sigma ))|$. Without loss of generality, we may assume that $N'$ is such that $|\Sigma (N')|$ is as small as possible. Moreover, we may assume without loss of generality that $N'$ and $N(\Sigma )$ are both maximal partially resolved. To obtain the required contradiction, we employ Corollary 2 to establish that $N'$ and $N(\Sigma )$ are isomorphic.

Since $\Sigma \subseteq \mathcal {I}(\Sigma )$ it is clear that $\mathcal {I}(\Sigma )$ contains all trivial splits of *X*. Furthermore, since $\Sigma$ is circular, Corollary 1(i) implies that $\mathcal {I}(\Sigma )$ is circular. Since $\mathcal {I}(\Sigma )$ is clearly $\mathcal {I}$-intersection closed and, by Property (Bi), $\mathcal {I}(\Sigma )$ is the split system Bu-displayed by $G(\mathcal {I}(\Sigma ))$ it follows that $\mathcal {I}(\Sigma )$ comprises all splits displayed by $N(\mathcal {I}(\Sigma ))$. Hence, by Corollary 2, up to isomorphism and partial resolution, $N(\mathcal {I}(\Sigma ))$ is the unique 1-nested network for which the displayed split system is $\mathcal {I}(\Sigma )$.

We claim that $\mathcal {I}(\Sigma )=\Sigma (N')$ holds too. By Corollary 1(iii), we have $\mathcal {I}(\Sigma )\subseteq \Sigma (N')$. To see the converse set inclusion assume that $S\in \mathcal {I}(\Sigma )$. Then *S* is either induced by (a) a cut edge of $N'$ or (b) *S* is not an m-split and there exists a cycle *C* of $N'$ that displays *S*. In case of (a) holding, $S\in \Sigma$ follows by the minimality of $|\Sigma (N')|$. So assume that (b) holds. Then there must exist some connected component $\Sigma _C\in \pi _0(\Sigma )$ that displays *S*. Hence, by Property (Bv), there exists some block $B_C\in \mathfrak {Bl}(\Sigma )$ such that the split system Bu-displayed by $B_C$ is $\Sigma _C$. Hence, $\Sigma _C$ is also displayed by $N(\Sigma )$. Since, as observed above, $\Sigma (N(\Sigma ))=\mathcal {I}(\Sigma )$ we also have $\Sigma (N')\subseteq \mathcal {I}(\Sigma )$ the claim follows.

(iii) Suppose *C* is a cycle of *N* and *v* is a vertex of *C*. Assume first that *v* is partially resolved. Then there exists a cut edge *e* of *N* that is incident with *v*. Note that the split $S_e$ displayed by *e* is also displayed by the two edges of *C* incident with *v*. In view of Property (Bi) and, implied by (Ci) and (Cii), that the cut edges of *N* are in 1–1 correspondence with the cut edges of $G(\Sigma )$, we obtain $S_e\in \Sigma$.

To see the converse assume that $e_1$ and $e_2$ are the two edges of *C* incident with *v* such that the split *S* displayed by $\{e_1,e_2\}$ is contained in $\Sigma$. Then *S* is compatible with all splits in $\Sigma -\{S\}$. By Property (Bv), it follows that there exists a cut edge *e* in $G(\Sigma )$ such that $S_e=S$. Combined with (Ci) and (Cii), it follows that *v* is partially resolved. $\square$

## Conclusion

Despite many years of research into rooted phylogenetic networks, our understanding of their combinatorial properties is still relatively poor limiting our ability to apply them within a biological context. To help make headway, uprooted versions of such networks have recently also been studied in the literature as they retain some of the biologically interesting properties of their rooted cousins. Here we call these types of networks uprooted phylogenetic networks and study them in terms of the split system they induce. Although our results are encouraging involving optimality results and a number of non-trivial characterizations, numerous questions that might be of interest have remained unanswered. For example, regarding Corollary 2, what is the minimal size of $\Sigma$ that allows one to, in our sense, uniquely recover $\Sigma (N)$? Also, is it possible to characterize split systems induced by more complex uprooted networks such as level-2 networks (i.e., networks obtained from level-1 networks by adding a cord to a cycle)?

Given that, from a combinatorial point of view, rooted phylogenetic networks are far less well understood than their unrooted counterparts, it might be interesting to investigate if uprooted networks could serve as some kind of intermediate structure to help bring to bear on them the rich body of literature for unrooted phylogenetic networks. For example, a number of reconstruction algorithms for rooted level-1 networks try to infer them from a collection of rooted binary phylogenetic trees on three leaves (Huson et al. 2010). Such trees are generally referred to as triplets, and in real biological studies, it is generally too much to hope for that a set of triplets contains *all* triplets induced by the (unknown) underlying network (see, e.g., Gambette et al 2017 for more on this). One way to overcome this problem is to employ triplet inference rules. Such rules are well known for rooted phylogenetic trees but are missing even for general rooted phylogenetic networks. The question therefore becomes if the work presented here combined with results on closures obtained in, for example, Gruenewald and Huber (2008) for unrooted phylogenetic networks might provide a starting point for developing such rules.

Finally, it is straightforward to check that rooted 1-nested networks are special cases of stable networks (see Gambette and Huber 2012 for the special case that the network is level-1) introduced in Huber et al. (2016) and that the later were linked with the gene tree and a species tree reconciliation problem in Huber et al. (2016), it might be interesting to explore if our arguments also help shed new light into that problem.

## Footnotes

1 We refer to Sect. 5 for a construction of such a network.
